# Alternative Targets for Modulators of Mitochondrial Potassium Channels

**DOI:** 10.3390/molecules27010299

**Published:** 2022-01-04

**Authors:** Antoni Wrzosek, Shur Gałecka, Monika Żochowska, Anna Olszewska, Bogusz Kulawiak

**Affiliations:** 1 Laboratory of Intracellular Ion Channels, Nencki Institute of Experimental Biology, Polish Academy of Sciences, 02-093 Warsaw, Poland; a.wrzosek@nencki.edu.pl (A.W.); s.galecka@nencki.edu.pl (S.G.); m.zochowska@nencki.edu.pl (M.Ż.); 2 Department of Histology, Medical University of Gdansk, 1a Debinki, 80-211 Gdansk, Poland; anna.olszewska@gumed.edu.pl

**Keywords:** mitochondrial potassium channels, cytoprotection, mitochondria, potassium channel openers

## Abstract

Mitochondrial potassium channels control potassium influx into the mitochondrial matrix and thus regulate mitochondrial membrane potential, volume, respiration, and synthesis of reactive oxygen species (ROS). It has been found that pharmacological activation of mitochondrial potassium channels during ischemia/reperfusion (I/R) injury activates cytoprotective mechanisms resulting in increased cell survival. In cancer cells, the inhibition of these channels leads to increased cell death. Therefore, mitochondrial potassium channels are intriguing targets for the development of new pharmacological strategies. In most cases, however, the substances that modulate the mitochondrial potassium channels have a few alternative targets in the cell. This may result in unexpected or unwanted effects induced by these compounds. In our review, we briefly present the various classes of mitochondrial potassium (mitoK) channels and describe the chemical compounds that modulate their activity. We also describe examples of the multidirectional activity of the activators and inhibitors of mitochondrial potassium channels.

## 1. Introduction

Earlier studies on isolated mitochondria have indicated that the transport of potassium ions is an important element in regulating their volume. This potassium transport process has also been observed in various cellular systems. Western blot analysis of isolated mitochondria has shown the presence of protein-binding antibodies specific to potassium channel counterparts in the plasma membrane. Finally, the presence of the mitoK channels in the inner mitochondrial membrane (IMM) was confirmed by measuring the activity of a single channel by the patch clamp technique. Mitochondrial potassium channels play an important role in mitochondrial functioning due to the flow of positive charge (in the form of K^+^) through the IMM. These channel proteins have been identified in the mitochondria of various tissues, including the heart, skeletal muscle, and neuronal cells. Activation of mitoK channels by passing K^+^ through the IMM causes changes in the mitochondrial membrane potential that increase mitochondrial oxygen consumption and influence mitochondrial ROS synthesis. Therefore, mitoK channels are an important part of cell signaling pathways and can be thought of as triggers for cell survival or cell death [1,2,3].

Several potassium channels have been identified in the IMM, such as ATP-sensitive potassium channels (mitoK_ATP_); small (mitoSK_Ca_), intermediate (mitoIK_Ca_), and large conductance (mitoBK_Ca_) calcium activated potassium channels; sodium dependent potassium (mitoSlo2) channels; and voltage-dependent (mitoKv1.3 and mitoKv7.4) potassium channels [4,5]. 

**Figure 1:**
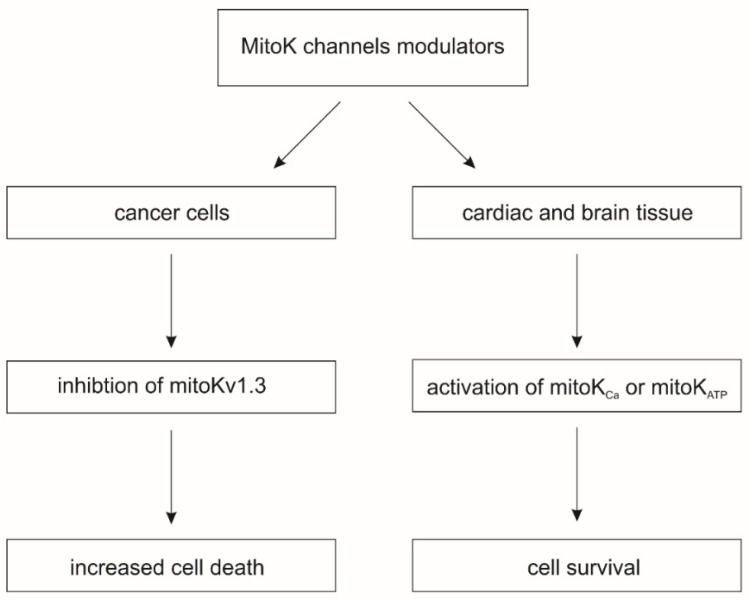
Diagram of the possible beneficial effects of activation or inhibition of mitoK channels with pharmacological substances.

The activation of mitoK channels by means of small molecule potassium channel openers (KCOs) induces cardio- and neuroprotective effects against various injuries, including ischemia-reperfusion. On the other hand, the pharmacological inhibition of mitoKv channels induces cell death in cancer cells (Figure 1) [1]. The beneficial effects induced by modulators of mitoK channels are related to changes in mitochondrial physiology. The activity of mitoK channels regulates the synthesis of mitochondrial ROS and mitochondrial matrix Ca^2+^ uptake, which directly regulates the opening of the mitochondrial permeability transition pore (mPTP), a key trigger of cell death pathways [6].

For these reasons, mitoK channels are promising targets for the development of new pharmacological tools to modulate their activity. However, the use of pharmacological modulators of mitoK channels carries a high risk that these compounds will interact with other targets in the cell, which can lead to unwanted effects or make it difficult to distinguish which effects are related to mitoK channels. In this review, we will provide basic information about the key modulators of mitoK channels and their alternative sites of action.

## 2. Modulators of Mitochondrial ATP-Sensitive Potassium Channels

The first potassium channel identified in the IMM was the ATP-sensitive potassium channel in rat livers [7]. This channel is inhibited by ATP and the antidiabetic sulfonylurea, glibenclamide [7]. Later, the same type of channel was identified in other tissues, such as the heart [8,9,10], brain [11,12,13], skeletal muscle [14], human T-lymphocytes [15], and skin fibroblasts [16]. Electrophysiological experiments revealed that the conductance of the channel is usually close to 100 pS [11,17,18,19]; however, channels with lower conductance, close to 10 pS, have also been reported [7,8]. These differences in reported conductance may result from the various experimental conditions. 

Initial findings suggested that the channel was formed by the Kir6.1 or Kir6.2 subunits, yet the molecular identity of mitoK_ATP_ remained unclear [20]. However, later studies showed that the ROMK2 isoform of the renal outer medullar potassium channel could be the structural component of the channel [9,21]. Interestingly, it was also suggested that the subunits of ATP synthase could form channels with mitoK_ATP_ pharmacological properties [22]. Recently, it was shown that the pore-forming subunit of the mitoK_ATP_ channel is a product of the CCDC51 gene [10]. Many studies have demonstrated that mitoK_ATP_ is inhibited by glibenclamide; therefore, it was speculated that the SUR subunit, which is a glibenclamide receptor, is an integral part of the channel. Indeed, CCDC51 interacts with mitoSUR, encoded by the ABCB8 gene. The channel formed by these two proteins has the canonical pharmacological properties of the mitoK_ATP_ channel [10]. Activation of the mitoK_ATP_ channel induces or mediates cardio- or neuroprotection against various insults, including I/R. This phenomenon is well documented; however, the details of the cytoprotection mechanism are still not fully understood [17,23,24]. 

The list of pharmacological modulators of the mitoK_ATP_ channel is relatively long. Diazoxide and BMS191095 can be treated as canonical pharmacological activators of the mitoK_ATP_ channel [25], along with nicorandil, cromakalim, pinacidil, or P1075. Inhibitors of the mitoK_ATP_ channel include glibenclamide (glyburide), 5-hydroxydecanoic acid (5-HD), tetraphenylphosphonium, and 4-aminopyridine [1,25]. 5-HD is described as a relatively selective inhibitor of mitoK_ATP_ channels, and it does not inhibit plasma membrane K_ATP_ channels [6,23]. However, all these compounds either have well-described alternative targets in the cell or induce cellular processes, which makes it difficult to reliably assess the detailed mechanism of their operation [26]. Importantly, most of these compounds were tested in the mitochondrial context. 

The most notable opener of mitoK_ATP_ channels is diazoxide, which is used in the treatment of diabetes [27]. Early studies revealed that this compound shows higher specificity toward mitoK_ATP_ than to K_ATP_ channels from the plasma membrane and sarcoplasmic reticulum [28]. Therefore, it is believed that the application of diazoxide at appropriately low concentrations should result in selective activation of the mitoK_ATP_ channel. However, diazoxide inhibits the activity of respiratory chain complex II [29]. Moreover, diazoxide directly activates protein kinase c epsilon (PKC-ε) (Table 1) [30]. Interestingly, it was shown that diazoxide and glibenclamide modulate the channel formed by CCDC51 only in the presence of the mitoSUR subunit [10]. Activation of ROMK2 found in mitochondria by diazoxide has also been observed [21]. 

Initial studies on isolated mitochondria suggested that BMS191095 was a specific activator of mitoK_ATP_ channels [31,32] that activated the channels in the nanomolar range of concentrations [32]. Electrophysiological studies confirmed that this compound opens cardiac, neuronal, and dermal fibroblast mitoK_ATP_ channels [11,16,33]. Several studies have shown that BMS191095 induces cytoprotection by activating mitoK_ATP_ channels. For example, BMS191095 induced cytoprotection against I/R injury in mice and rat cardiac tissue [32,34] and pig skeletal muscle [35]; cytoprotection induced by this compound was reversed by 5-HD. Additionally, BMS191095 was shown to activate neuronal mitoK_ATP_, regulate neuronal mitochondrial function [36,37], and induce neuroprotection against various insults [36,38,39]. It was also shown that BMS191095 and the less-selective BMS180448 inhibited human platelet aggregation; preincubation with both glyburide and 5-HD blocked this effect [40]. In contrast, the application of BMS191095 protected myoblast C2C12 cells against toxicity induced by the deregulation of calcium homeostasis, and these effects were not reversed by 5-HD [41]. This compound was also shown to induce neurotoxicity effects [24,32]. 

Nicorandil, like diazoxide, was shown to mimic ischemic preconditioning of heart tissue by the activation of mitoK_ATP_ channels [42,43]. However, this compound can also activate sarcolemmal K_ATP_ channels [44]. Other studies have reported that nicorandil can be a donor of nitric oxide (NO) [45,46]. Additionally, it can stimulate superoxide dismutase and inhibit xanthine oxidase activity, therefore acting as an antioxidant [46,47,48]. The bidirectional effects of nicorandil were recently observed in a study describing the beneficial effects of this compound on fatigue in slow skeletal muscle fibers [43]. On the one hand, nicorandil activated the channel; on the other hand, it acted as an antioxidant and NO donor [43]. Further experiments revealed that nicorandil blocks mitochondrial glutamate malate-driven respiration in skeletal muscle [49]. Nicorandil can induce cytoprotection in dystrophin-deficient cardiomyocytes, and these effects are only partially reversed by the mitoK_ATP_ blocker 5-HD [50]. Another recent example of the bidirectional activity of nicorandil can be seen in the regulation of the expression of selected genes, such as heme oxygenase-1 or interleukin-8, by nicorandil in human umbilical vein endothelial cells (HUVECs). Only some of the observed effects were reversed by 5-HD, and thus were linked to mitoK_ATP_ activity [51]. In addition to the other openers listed above, it has also been suggested that malonate and atpenin A5, which are inhibitors of complex II, can activate mitoK_ATP_ channels and induce cardioprotection [52,53].

Similar to mitoK_ATP_ openers, inhibitors of this channel can either interact with other proteins or enter various metabolic pathways. For example, 5-HD, which is a canonical mitoK_ATP_ inhibitor, was shown to inhibit sarcolemmal K_ATP_ channels [54]. In contrast, 5-HD does not inhibit mitoK_ATP_ from rat brains [11]. It was also found that 5-HD is metabolized by acyl-CoA synthetase and enters beta-oxidation, which can induce the switch from glucose to fatty acid metabolism [55,56,57]. Therefore, the direct interaction of 5-HD with mitoK_ATP_ has been questioned. Additionally, 500 μM of 5-HD was shown to stimulate the activity of mitochondrial complex II and inhibit complex III in skeletal muscle cells [49]. Another study revealed that both glibenclamide and 5-HD can interact with ADP/ATP carriers from the inner mitochondrial membrane [58]. Additionally, glibenclamide can activate the mitochondrial permeability transition pore by increasing its sensitivity to calcium ions [59]. Moreover, glibenclamide inhibits chloride channels [60,61]. 

To examine whether the ROMK protein acts as a possible mitoK_ATP_ channel-forming component, research has focused on the use of compounds regulating the activity of this protein. For example, it was reported that VU591 inhibits ROMK channels with high specificity [62,63,64]. However, this molecule induced mitochondrial depolarization and matrix contraction in mitochondria from both wild-type and ROMK knockout cells. [65]. Moreover, VU591 reversed matrix swelling induced by BMS191095 in the ROMK knockout cells. These observations suggest that VU591 has some alternative targets in mitochondria [65].

**Table 1:** Examples of mitoK_ATP_ channel modulators and their off-target activity.

Name	Function	Example of Off-Target Activity	Ref.
Diazoxide	Opener	Inhibition of mitochondrial complex II. Protonophoric properties. PKC-ε activator. ROS inducer.	[16,29,30,37,57]
BMS191095	Opener	Induction of mitoK_ATP_-independent cytoprotection. Induction of neurotoxicity.	[16,24,32,37,41]
Nicorandil	Opener	NO donor. Antioxidant, inhibition of xanthine oxidase activity.	[43,45,46,47]
5-HD	Inhibitor	Sarcolemmal K_ATP_ channel inhibitor. Substrate for acyl-CoA synthetase. Stimulation of mitochondrial complex II. Inhibition of mitochondrial complex III. Possible interaction with mitochondrial ADP/ATP carriers.	[49,54,55,56]
Glibenclamide	Inhibitor	Activation of mPTP. Possible interaction with mitochondrial ADP/ATP carriers. Inhibitor of cAMP-activated chloride channels.	[58,59,60,61]
VU591	Inhibitor (ROMK)	General inhibitor of ROMK channels. Mitochondria uncoupling.	[64,65]

## 3. Mitochondrial Calcium-Activated Potassium Channels

In the IMM, a group of calcium-activated potassium channels have been identified, namely large conductance K^+^ (mitoBK_Ca_) channels [66], intermediate conductance K^+^ (mitoIK_Ca_) channels [67], and small conductance K^+^ (mitoSK_Ca_) channels [68]. Their common property is regulation by calcium ions—their open probability increases in the presence of Ca^2+^ [17]. This common activation mechanism may indicate that these proteins are activated under the same physiological conditions. However, these channels differ in terms of other regulatory mechanisms. In addition, a different spectrum of pharmacological substances regulates their activity. 

### 3.1. Large Conductance Calcium-Activated Potassium Channels

The mitoBK_Ca_ channel was originally identified in the mitochondria of LN229 glioma cells by the patch clamp technique. The recorded channel had a conductance of 295 pS measured in a 150 mM KCl bath and pipette solution [66]. Later, the channel was described in other tissues, including the heart [69], brain [70,71], skeletal muscle [72], endothelium [73], dermal fibroblasts [74], and pulmonary and kidney epithelial cells [75,76]. Interestingly, similar channels have been identified in the mitochondria of mammals, lower organisms, and plants [77,78,79,80]. 

The pore-forming α subunit of both the mitoBK_Ca_ and the plasma membrane BK_Ca_ channels is encoded by the KCNMA1 gene. The VEDEC isoform of the α subunit (the name comes from the amino acid sequence at the C-terminus of the protein) is targeted to cardiac mitochondria [81,82]. Recently, it was shown that transfection with a VEDEC-encoding plasmid results in mitoBK_Ca_ channel activity in HEK293T cells [83].

Similar to their plasma membrane counterparts, mitoBK_Ca_ channels are modulated by various endogenous modulators. It was shown that heme and hemin inhibit channel activity, and that carbon monoxide reactivates channels blocked by heme [84,85,86]. The channel is also modulated by other gasotransmitters, such as hydrogen sulfide (H_2_S) [87,88,89,90,91,92], redox signals, and protons [93]. The channel is also activated by 17β-estradiol, which can lead to cardioprotection [94]. 

Several synthetic modulators have been used to activate the mitoBK_Ca_ channel [6,95]. 

**Figure 2:**
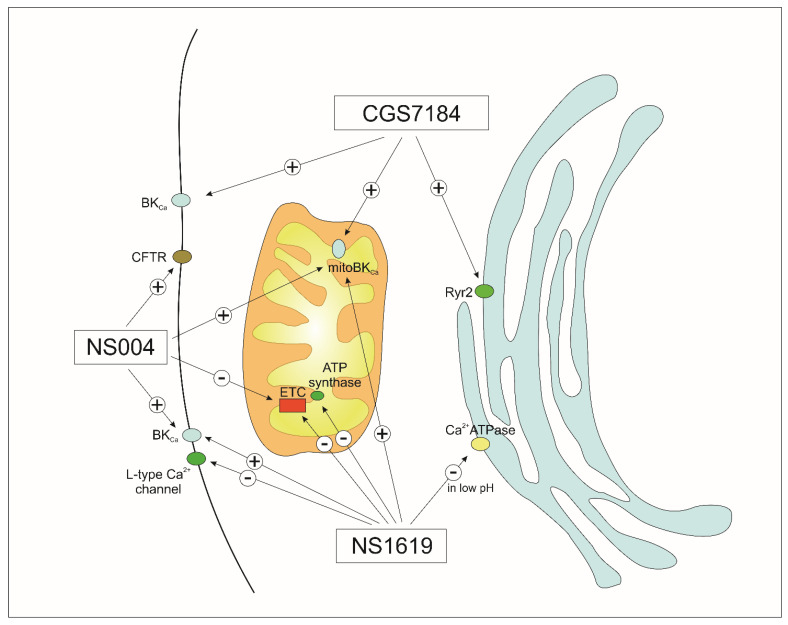
Scheme of the targets of mitoBK_Ca_ openers NS1619, NS004, and CGS7184. The targets located in mitochondria, endoplasmic reticulum, and plasma membrane are shown. “+”—activation, “−“—inhibition.

Some of the first synthetic BK_Ca_ channel activators used for mitoBK_Ca_ channels were the benzimidazolone derivatives NS1619 and NS004 [69,96,97]. Interestingly, NS004 was also described as a cystic fibrosis transmembrane conductance regulator (CFTR) channel opener [98]. Application of NS1619 protects the heart tissue from I/R injury, and many studies have shown that this effect may be mediated by mitoBK_Ca_ [69,82,99]. NS1619 acts by shifting the voltage-sensor of BK_Ca_ channels toward the activated state, as the open probability is barely altered when the voltage sensing domain (VSD) is at rest [100,101]. Although NS1619 is a known mitoBK_Ca_ channel opener, this compound has many effects unrelated to the channel. For example, it can induce nonselective ion transport across the inner mitochondrial membrane [102] and inhibit complexes of the respiratory chain [102,103]. Another study showed that NS1619 has oligomycin-like properties and inhibits mitochondrial ATP synthase. Additionally, NS1619 inhibits sarco/endoplasmic reticulum Ca^2+^-ATPase (SERCA) [104], and L-type calcium channels (Figure 2) [105]. NS1619 also stimulates Ca^2+^-gated chloride currents in smooth muscle cells of the rabbit pulmonary artery [106]. BK_Ca_ channel-independent immediate and delayed preconditioning of neuronal cells was also observed after the application of NS1619 [107,108]. 

NS11021, a biarylthiourea derivative, has a higher potency and selectivity as a BK_Ca_ channel opener than NS1619 [109]. Application of NS11021 shifts the voltage-activation curve of the channel to more negative potentials, and activation is independent of free Ca^2+^ concentration [100,110,111]. NS11021 increases the open probability of BK_Ca_ channels by altering the gating kinetics without affecting the single-channel conductance. It also protects the heart from I/R injury via mitoBK_Ca_ channel activation [110]. Interestingly, NS11021-induced activation of mitoBK_Ca_ channels prevents cold-storage induced injury of kidney epithelial cells [75].

Although NS11021 was thought to be more specific than NS1619, it was later shown to induce mitochondrial depolarization in a sucrose medium in the absence of K^+^ salt [112]. It was also found that NS11021 applied at higher concentrations (10–30 μM) can activate K_V_7.4 and have minor inhibitory effects on Kv7.2/7.3 channels. The same study showed that NS11021 is a positive modulator of α7 nicotinic acetylcholine receptors at concentrations of 10–30 µM [109]. 

Another set of mitoBK_Ca_ channel openers is CGS7181 and CGS7184. Mitoplast patch clamp recording has revealed that the mitoBK_Ca_ channel of glioma cells is activated by CGS7184 [113]. Application of this opener decreased ROS synthesis by brain mitochondria in a model of reversed electron flow, and this effect was reversed by mitoBK_Ca_ channel inhibitors [114]. However, in contrast to the effects of NS1619, CGS7184 and its derivative CGS7181 induced cell death, an effect unrelated to mitoBK_Ca_ channel activation [113,115]. CGS7184 can also directly activate ryanodine receptor (RyR2) calcium release [116]. Evidently, the modulation of alternative targets by these compounds may be responsible for the observed increase in cytosolic calcium ion concentration [95]. 

Interestingly, both CGS7184 and NS1619 show mitoBK_Ca_ channel activation in single channel patch clamp technique measurements; however, they exert opposing effects on cells. CGS7184 has a cytotoxic effect while NS1619 is cytoprotective. Therefore, the cytotoxic outcome of CGS7184 must be related to the off-target effects of this compound. Conversely, in the case of NS1619, the off-target effect is synergistic to some extent, leading to cytoprotection, especially in I/R processes. One of the mechanisms of action that distinguishes these compounds is the different manner in which calcium ions are released from the endoplasmic reticulum. CGS7184 releases Ca^2+^ by opening RyR2 channels, while NS1619 releases Ca^2+^ through the inhibition of SERCA in a pH-dependent manner [117,118]. Opening of RyR2 channels by CGS7184 naturally activates SERCA by Ca^2+^, which leads to a significant increase in the consumption of intracellular ATP, while NS1619 causes inhibition of SERCA, reducing ATP consumption. NS1619′s inhibition of SERCA increases as pH decreases and is reversible. Notably, a drop in pH occurs during ischemia (Figure 2).

Another interesting mitoBK_Ca_ channel modulator is chlorzoxazone, which is an FDA-approved centrally acting muscle relaxant [119]. This compound produces a left shift in the activation curve of BK_Ca_ channels, yet it does not affect the Ca^2+^-sensitivity of the channels during that process [119]. It was used to treat a patient with progressive cerebellar degeneration, who had a BK_G354S_ mutation in the KCNMA1 gene [120]. Chlorzoxazone also activates SK_Ca_ and IK_Ca_ channels, and 30 μM chlorzoxazone suppresses voltage-dependent L-type Ca^2+^ currents [119]. Another mitoBK_Ca_ channel modulator is a synthetic inhibitor with a natural origin: diCl-DHAA (12,14-dichlorodehydroabietic acid). It was reported to reduce I/R in rat cardiac myocytes [121,122]. 

The mitoBK_Ca_ channel is also activated by naturally occurring openers. Similar to the plasma membrane BK_Ca_ channel, the mitoBK_Ca_ channel is activated by the sex hormone 17-estradiol (17β-estradiol, EST). 17β -estradiol has been applied to the mitoBK_Ca_ channels in vascular smooth muscle using the black lipid membrane technique or the ventricular mitoplasts of rats using patch clamp [94]. The activation of mitoBK_Ca_ channels by 17β-estradiol requires the β1 subunit [123]. 17β-estradiol has been described as an L-type channel agonist that induces a rapid increase in intracellular calcium concentration through the potentiation of the channel and the activation of cascade signaling pathways, such as Src/ERK/CREB/Bcl-2 [124]. Another example of a naturally occurring mitoBK_Ca_ channel modulator is naringenin, a plant-derived flavonoid found in a variety of fruits and herbs [125]. Naringenin has been found to induce cardioprotection by activating mitoBK_Ca_ channels [126]. Recently, it was shown that naringenin also activates mitoBK_Ca_ and mitoK_ATP_ channels in skin fibroblasts [127]. Naringenin also has a stimulatory effect on BK_Ca_ channels in the absence of the auxiliary β subunits of the channel, increasing the probability of channel opening [128]. The beneficial effects of naringenin have been described for many disorders, including cardiovascular, pulmonary, metabolic, and neurological issues [95]. When applied to NSC-34 neuronal cells, naringenin shifted the activation curve of the M-type K^+^ current (Kv7.2, 7.3, and 7.5) to more negative potentials [128]. 

Several inhibitors of mitoBK_Ca_ channels have been described. This group contains the short peptides charybdotoxin (ChTx) and iberiotoxin (IbTx), quinine, and diterpene paxilline. Charybdotoxin is a scorpion (*Leiurus quinquestriatus*) toxin identified in 1985 as a BK_Ca_ channel blocker [66,129,130,131]. Later, it was also shown to inhibit mitoBK_Ca_ channels [66]. In addition, charybdotoxin also inhibits the IK_Ca_, Kv1.2, and Kv1.3 channels and, at lower potency, the Kv1.6 channels [132].

Iberiotoxin, a toxin from the red scorpion *Buthus tamulus* is 68% homologous to charybdotoxin; however, it does not block any other ChTX-sensitive K^+^ channels [133,134]. Moreover, iberiotoxin can block the BK_Ca_ channel in the presence of the auxiliary β1 subunit [135]. Interestingly, both charybdotoxin and iberiotoxin are inactive in the presence of the auxiliary β4 subunit [136,137].

Paxilline, a tremorgenic indole toxic alkaloid produced by *Penicillium paxilli*, has been used to block mitoBK_Ca_ channels in guinea pig ventricular cells, rat ventricular myocytes, and rat heart and liver cells [138,139]. Higher concentrations of paxilline modulate SERCA at the phosphoenzyme level [140,141]. At a concentration of 10–50 µM, paxilline decreases light scattering, slightly uncouples respiration [142], and protects HT22 cells against glutamate-induced cytotoxicity [143].

Lastly, quinine is a compound from cinchona trees that has been found to inhibit BK_Ca_ channel-caused K^+^ uptake into isolated mitochondria. Quinine is also known to inhibit K_V_2.2 and K2P18.1 channels [144]. Structural studies have shown that BK_Ca_ channels possess a conserved heme-binding sequence motif. Hemin binds to the linkage segment between the RCK1 and RCK2 domains and induces conformational changes different than those induced by Ca^2+^ [85]. It regulates the inactivation of the K^+^ channel and N-type inactivation of Kv1.4 and Kv3.4 channels [145].

### 3.2. Intermediate Conductance Calcium-Activated Potassium Channels

Intermediate conductance calcium-activated potassium channels from the mitochondrial inner membrane show a conductance close to 27 pS and were identified for the first time in human colon cancer cells [67]. The pore-forming subunit of IK_Ca_ channels from the plasma membrane has six transmembrane spanning regions (S1–S6), with the conducting pore located between S5 and S6. The gating is conferred upon Ca^2+^ binding to calmodulin (CaM), which is constitutively bound to the C-terminus of each channel subunit [146,147]. It was shown that the activity of IK_Ca_ channels in the mitochondria and plasma membrane influences oxidative phosphorylation in pancreatic ductal adenocarcinoma cells [148]. 

The IK_Ca_ channels from the plasma membrane and mitochondria are activated by several synthetic modulators, such as NS309 [148,149,150,151,152,153], DCEBIO [150,154,155,156], and riluzole (Table 2). Riluzole has not been tested on mitoIK_Ca_ channels per se, though it was used to measure mitochondrial membrane potential (Δψ). However, these compounds are not selective modulators of IK_Ca_ channels, as they can also activate SK_Ca_ channels [157]. Riluzole is an FDA-approved drug [154] that activates K2P10.1, TRPC5 [158], K2P2.1, K2P4.1, and Slo2 channels [159]. This compound can also inhibit Na^+^, Trpm4 [160,161,162,163], and Cl^-^ channels [164], as well as GABA reuptake [165]. Moreover, riluzole increases glutamate uptake and modulates glutamate receptors [166]. Interestingly, NS309 is believed to be a more potent activator of the IK_Ca_ channel than 1-EBIO or DCEBIO [149]. 

IK_Ca_ channels are inhibited by clotrimazole [67,155,167,168,169], which can also inhibit BK_Ca_ channels [170]. Clotrimazole plays a role in the acute inhibition and chronic induction of human cytochrome P450-dependent enzymes, leading to liver damage. The second inhibitor of the channel, TRAM-34, is a clotrimazole-based derivative [67,148,155,171,172]. It is more selective for IK_Ca_ channels than it is for K_V_, BK_Ca_, and SK_Ca_ channels [173]. TRAM-34 decreased astrogliosis and microglial activation, and attenuated memory loss in an Alzheimer’s disease mouse model [174].

**Table 2:** Examples of mitoIK channel modulators and their off-target activity.

Name	Function	Example of Off-Target Activity	Ref.
Riluzole	Opener	Activation of SK_Ca_, K2P2.1, K2P10.1, K2P4.1, TRPC5, Slo2 channels. Inhibition of Trpm4 and chloride channels.	[161,162,164,167]
NS309	Opener	Opener of SK_Ca_ channels.	[149,151]
DCEBIO	Opener	Opener of SK_Ca_ channels.	[153,159,160]
Clotrimazol	Inhibitor	Inhibition of BK_Ca_ channels. Inhibition of cytochrome P450-dependent enzymes.	[67,170]
TRAM-34	Inhibitor	Inhibition of Kv, BK_Ca_, SK_Ca_ channels when applied at higher concentration.	[150,173]

### 3.3. Small Conductance Calcium-Activated Potassium Channels

In the inner mitochondrial membrane, small conductance calcium-activated potassium channels have also been identified [68]. The conductance of SK_Ca_ channels is approximately 4–14 pS. The gating of these channels is conferred by Ca^2+^ binding to calmodulin, which, as with in IK_Ca_ channels, is constitutively bound to the C-terminus of each channel subunit [146,147]. The plasma membrane and mitochondrial SK_Ca_ channels are activated by several compounds, such as 1-EBIO, DCEBIO, NS309, CyPPA, or riluzole [151,155,175,176,177]. As mentioned above, these compounds can also activate IK_Ca_ channels; however, not all of these compounds were used in mitoSK_Ca_/IK_Ca_ channel studies. The activation of the mitoSK_Ca_ channel with NS309 is known to reduce neurotoxicity [151]. NS309 is a more potent activator of the recombinant SK2 channels than DCEBIO and 1-EBIO [178]. It has also been shown that 1-EBIO activates CFTR channels [179]. CyPPA-mediated activation of the mitoSK2 channel leads to neuroprotection of HT-22 cells [177,180]. CyPPA can also inhibit melanogenesis by modulating the GSK3β/β-catenin/MITF pathway, as well as reduce nitric oxide release in microglia [181]. 

The mitoSK_Ca_ channel is inhibited by a canonical SK_Ca_ channel peptide blocker, apamin [151,155,182]. This inhibitor blocks SK_Ca_ channels in concentrations ranging from pM to nM. Apamin is known to bind to the outer pore region of the SK_Ca_ channel; however, it is membrane impermeable and can show off-target activity at higher concentrations. Apamin regulates gene expression in various signaling pathways [183]; for example, it downregulates or inhibits the expression of TNF-α, intracellular cell adhesion molecule (ICAM)-1, vascular cell adhesion molecule (VCAM)-1, transforming growth factor (TGF)-β1, fibronectin, the NF-κB signaling pathway, and signal transducers and activators of transcription (STAT) in vitro, thereby inhibiting proinflammatory cytokines and type 2 helper (Th2) lymphocyte chemokines [184].

The mitoSK_Ca_ channel, similar to its plasma membrane counterpart, can be blocked by several synthetic inhibitors, such as NS8593 or UCL1684 [182,185]. UCL1684 mimics the binding residues of the structural elements of a naturally occurring inhibitory neurotoxin: apamin. This compound displaces apamin binding and is considered a pore blocker, acting at the apamin binding site [186]. SK_Ca_ channels are also blocked by chlorzoxazone [187,188,189]. This compound, which serves as a muscle-relaxing drug and a probe for human liver cytochrome P-450IIE1 (CYP2E1), suppresses voltage-dependent L-type Ca^2+^ current. 

## 4. Mitochondrial Voltage-Dependent Potassium Channels

In the inner mitochondrial membrane, voltage-gated potassium channels, including mitoKv1.3, mitoKv1.5, and mitoKv7.4, have been identified [1]. These channels have also been found in the plasma membrane. Interestingly, the Kv1.3 channel is also present in the endoplasmic reticulum and the Golgi apparatus. The activity of Kv channels is regulated by changes in the cell membrane potential [190]. These channels are composed of voltage-sensing and pore-forming α-subunits and auxiliary β-subunits that enable them to perform a wide variety of physiological functions [191]. Each α-subunit is composed of six transmembrane domains (S1–S6) with the S1–S4 transmembrane domains surrounding a central pore domain built of two S5–S6 helices. The four α-subunits link together to form the functional square-pore structure of the Kv channel [192]. Previous work has shown that mitoKv1.3 channels are highly overexpressed in cancer cells and immune cells, and that modulation of these channels is effective in the treatment of cancer [193]. There are many Kv channel modulators, and we will focus only on those modulators that have been described as acting on the Kv channels found in mitochondria. Most of these modulators also modulate Kv channels present in various cellular compartments with different specificity dependent on the concentration used.

### 4.1. Mitochondrial Kv1.3 and Kv1.5 Channels and Their Modulators

The pore-forming subunit of the Kv1.3 channel is encoded by the KCNA3 gene. The presence of mitoKv1.3 channels was originally observed in T cells [194]; however, mitoKv1.3 channels are also present in cancer cells, such as leukemia Jurkat T cells, prostate cancer PC-3 cells, and breast cancer MCF-7 cells [195]. The Kv1.3 channel is also found in the nuclear membrane of Jurkat T cells, MCF-7 cells, A549 lung cancer cells, and SNU-484 gastric cancer cells, as well as in human brain tissue [196]. The mitoKv1.5 channel has been found in the inner mitochondrial membrane of J774 macrophages [197].

Kv1.3 channels have been identified in mitochondria by incubating isolated mitochondria with plasma membrane Kv1.3 channel-specific inhibitors, such as margatoxin (MgTx) and *Stichodactyla* toxin (ShK). These compounds are effective in inducing hyperpolarization of the mitochondrial membrane [194]. 

**Figure 3:**
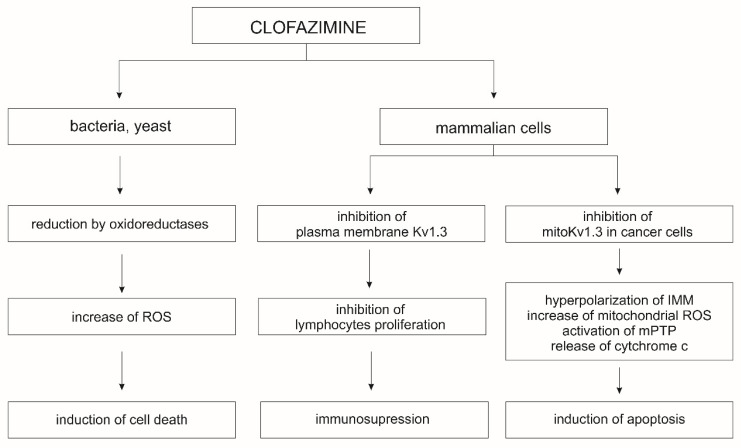
Diagram showing the multidirectional effects of clofazimine.

The Kv1.3 and Kv1.5 channels of the plasma membrane play a key role in the regulation of cell proliferation, migration, and differentiation; their inhibition leads to blockage of the cell cycle and cell proliferation [198]. In contrast, the inhibition of mitoKv1.3 and mitoKv1.5 channels, either by the Bax protein or by specific inhibitors, plays a key role in the activation of the apoptotic pathway [195,197]. Other known inhibitors of the mitoKv1.3 channel are clofazimine and the non-phototoxic 5-methoxy-psoralen derivatives, Psora-4 and PAP-1 [199]. Application of MgTx, ShK, or Psora-4, results in changes in membrane potential, synthesis of reactive oxygen species (ROS), and release of cytochrome *c* from the mitochondrial intramembrane space [200]. Both MgTx and ShK act directly on the mitoKv1.3 channel when applied to mitoplasts. However, these inhibitors are peptides, which limits their permeation of cellular membranes; therefore, they are not useful as mitoKv1.3 channel inhibitors in vivo and in whole-cell studies. The peptide nature of these blockers also limits their ability to block channels in the plasma membrane, and their long-term administration carries the risk of provoking an immune response. This is not the case with psoralen derivatives, such as Psora-4 and PAP-1, and clofazimine, an antibacterial drug used in the treatment of leprosy (Table 3). Clofazimine inhibits the mitoKv1.3 and mitoKv1.5 channels, making it highly effective for studies on cancer treatment. In Vivo studies on the murine B16-F10 orthotopic melanoma model have demonstrated that the application of clofazimine resulted in a 90% reduction in tumor mass (Figure 3) [197].

In mice transplanted with orthotopic human COLO 357 cells, intraperitoneal administration of clofazimine resulted in a 50% reduction in tumor mass [201]. The use of clofazimine also led to apoptosis of B cells in patients suffering from chronic lymphocytic leukemia [199]. Additionally, clofazimine inhibits in vitro Wnt signaling in a wide range of triple-negative breast cancer subtypes that do not respond to hormonal drugs and targeted therapies [202]. Clofazimine also has an immunosuppressive effect that results from the selective blocking of the Kv1.3 channel, which is involved in the proliferation and survival of T effector cells, and, therefore, is a good target for the treatment of autoimmune diseases. Blocking the Kv1.3 channel changes the amplitude and frequency of intracellular Ca^2+^ oscillation and inhibits the calcineurin/nuclear factor of activated T-cells (NFAT) signaling pathway [203]. Clofazimine interacts with and eventually blocks Kv1.3 channels in a precise and state-dependent manner. It may block open Kv1.3 channels during long depolarizations or block inactivated channels once they have been opened by brief depolarizations. Clofazimine’s mechanism of blocking plasma membrane Kv1.3 channels opens up its possibility for use in the treatment of autoimmune diseases, where the expression of these channels in immune cells is increased [204]. Clofazimine is also a promising drug for the treatment of cryptosporidiosis and nonviral diarrhea in children [205]. In addition, it was effective at preventing skin graft rejection in a mouse transplant model [203]. The use of clofazimine in the treatment of drug-resistant tuberculosis has also been described. The mechanism of action in this case is not entirely clear; however, most likely it acts on *Mycobacterium tuberculosis* as a pro-drug that is reduced by NADH dehydrogenase, releasing reactive oxygen species upon reoxidation. Clofazimine likely competes with menaquinone (MK-4), a key cofactor in the mycobacterial electron transfer chain, for reduction by NADH [206]. 

Psora-4 has been shown to bind to the central, highly-conserved pore of Kv channels, and it may also bind to the side pockets formed by the backsides of the S5 and S6 helices and the S4–S5 linker [207]. The simultaneous occupation of both binding sites by the drug results in an extremely stable nonconducting state and increases Psora-4 selectivity for mitoKv1.3 and mitoKv1.5 channels [207]. Due to the inhibition of the plasma membrane Kv1.3 channel, Psora-4 can promote the differentiation and morphological, as well as electrophysiological, maturation of neurons derived from progenitor cells; therefore, it can be used in the treatment of multiple sclerosis [208]. By inhibiting the Kv1.3 channel present on T cells and glomerular infiltrating macrophages, Psora-4 halts rapidly progressive glomerulonephritis and may also be used to treat other autoimmune diseases [209].

PAP-1-MHEG is a recently synthetized PAP-1 derivative that has a higher degree of solubility due to the presence of an oligomeric chain of ethylene glycol. At a concentration of 10 µM, PAP-1-MHEG was able to induce apoptosis more effectively than could PAP-1 in cells isolated from leukemia patients that express mitoKv1.3. PAP-1-MHEG, similar to the other derivatives, blocks the mitoKv1.3 channel through the alkyl-O-phenyl part and causes depolarization of the mitochondrial membrane, which is crucial for the induction of apoptosis. It has also been found that the coumarin ring of PAP-1 and its derivatives acts as a “ROS generator” (Table 3) [210]. However, the unique aspect of PAP-1-MHEG is its ability to block the activity of respiratory chain complex I, via its the methoxy oligoethylene glycol (MOEG) domain, which may interfere with electron transport and the ubiquinone redox cycle. This interference is likely due to the proximity of the mitoKv1.3 channel and the NDUFS1 and NDUFS3 subunits of respiratory chain complex I; however, this requires further research [210]. None of the 5-methoxy-psoralen derivatives (Psora-4, PAP-1, PAPTP, PCARBTP, and PAP-1-MHEG) show any cytotoxic effect in cells with significantly fewer mitoKv1.3 channels, and do not significantly increase the mortality of tumor cells that overexpress mitoKv1.3 channels and have elevated ROS levels [199,210,211]. 

### 4.2. Mitochondrial Kv7.4 Channel and Its Modulators

The Kv7 channel subfamily consists of five members named sequentially from Kv7.1 to Kv7.5, encoded by the KCNQ1-5 genes. Each of these channels forms homotetramers, or sometimes heterotetramers, that exhibit different tissue distributions and physiological roles [212]. The Kv7.1 channel is present in the heart, pancreas, gastrointestinal tract, thyroid gland, brain, portal vein, and inner ear. The Kv7.2 and 7.3 channels are mainly expressed in the central nervous system; Kv7.4 is the major voltage-gated potassium channel in the inner ear and bladder smooth muscle; and Kv7.5 is expressed in skeletal muscle and plays a key role in contractility [213].

The presence of mitoKv7.4 channels was demonstrated in the mitochondria of H9c2 cardiomyoblasts and in isolated adult cardiomyocytes. This channel can be activated by retigabine and flupirtine, which induce mitochondrial depolarization and reduce the uptake of calcium ions into the matrix, resulting in cardioprotection against ischemia/reperfusion. This effect was abolished by the Kv7.4 channel inhibitor XE991 [214]. 

Both flupirtine and retigabine are activators of virtually all types of Kv7 channels, except Kv7.1, and thus have a broad spectrum of action and several side effects (Table 3).

Flupirtine is a triaminopyridine compound that serves as a nonopioid analgesic in the treatment of acute and chronic musculoskeletal pain [215]. It also has muscle relaxant [216] and anticonvulsant properties [217]. Flupirtine activates Kv7.2 and Kv7.3 channels, thus inhibiting the firing of sustained neuronal action potentials. This inhibits the excitability of neurons and reduces the excitatory transmission of neurotransmitters [218].

Flupirtine also acts as an N-methyl-D-aspartate (NMDA) antagonist and can reverse the effects of NMDA receptors, which play a key role in learning, although flupirtine does not bind directly to them. Additionally, flupirtine normalizes the level of intracellular glutathione and increases the expression of the antiapoptotic protein Bcl-2 in neuronal cells exposed to prion proteins. This neuroprotective effect of flupirtine can be used in the treatment of prion diseases [219]. Flupirtine has also been studied in the treatment of tinnitus and overactive bladder [220]. Approximately 8–12% of bioavailable flupirtine in humans is eliminated as a derivative of mercapturic acid by conjugating glutathione with intermediate quinone diimine, which is believed to be responsible for the hepatotoxicity of flupirtine [221]. 

Retigabine (RTG), also known as ezogabine, is used as an anticonvulsant drug (Table 3) [222]. This potassium channel opener has been approved as an adjunct to treat drug-resistant partial seizures [223]. However, due to its low benefit-to-risk ratio, the use of retigabine was discontinued in 2017, at which time the search for safer and more effective alternatives commenced [212].

RTG binds to Kv7.2-5 channels (encoded by KCNQ2-5) in neurons near the channel gate, which stabilizes the open channel state. The amino acid residues of glycine (G) 301 and tryptophan (W) 236 are crucial for this binding. The absence of W236 in KCNQ1 (which encodes the Kv7.1 channel) explains the lack of RTG binding to that channel [224]. Retigabine and flupirtine have shown antinociceptive effects in a rat model of gout. It is believed that Kv7 channels generate low-threshold, inactivating voltage-gated potassium currents that play an important role in regulating the excitability of nociceptive neurons [225]. To date, the analgesic effect of RTG has been demonstrated in models of pain due to neuroplasticity, bone cancer [226], inflammation [227], nerve degeneration [228], and the application of capsaicin to the viscera [229]. RTG at high concentrations may also result in positive allosteric modulation of γ-aminobutyric acid (GABA) receptors, specifically GABAA, and affect GABA metabolism (at concentrations > 10 µM). At high concentrations (>100 μM) RTG may also be a weak inhibitor of sodium and calcium channels [230]. RTG and ethanol show a similar effect on GABAergic and glutamatergic neurotransmission, which suggests they may interact. RTG reduces the basal rate of dopamine release in a dose-dependent manner in the mesolimbic system, which may be important in the treatment of psychotic diseases [231]. In contrast to RTG, high doses of ethanol increase dopaminergic neurotransmission [232]. Repeated administration of RTG significantly reduces neurological changes in the hippocampus caused by long-term administration of ethanol [233]. RTG also reduces NMDA-induced cell apoptosis in organotypic hippocampal cultures and in models of hypoxia and hypoglycemia [234]. A reduction in memory impairment and a slight improvement in learning post-alcohol consumption were also observed in rats treated with RTG [235]. There are few other reports on the effects of RTG on memory; however, one study has shown that even a single administration of RTG immediately after a traumatic experience can prevent fear memory consolidation and thus prevent posttraumatic stress disorder [236]. Retigabine has shown a sedative effect in studies of mania and bipolar disorder treatment [237]. It also significantly reduces the duration of in vitro myotonia, which causes excessive muscle stiffness, by activating K^+^ currents during the sequences of action potentials [238]. After cerebral ischemia and reperfusion, there was a reduction in the area of cerebral infarction, tightening of tight connections between the cells of the blood vessel epithelium, reduced permeability of the blood-brain barrier, and reduced levels of MMP-2 and MMP-9 proteins in the ischemic area under the influence of RTG [239]. RTG and flupirtine, due to their oxidizing properties, reduced serum-induced ROS levels in neurons in the dentate gyrus; thus, they showed neuroprotective effects unrelated to the activity of Kv7 channels [234]. At high concentrations (>100 μM), retigabine inhibits Kv1-9 and Kv11 channels; at low concentrations (0.3–3 µM) it also inhibits the Kv2.1 channel. This inhibition is partially irreversible and may account for some of the undesirable effects of RTG application. The neuroprotective effect of RTG in neurons may result from the coordinated action of RTG on the Kv2.1 and Kv7 channels [240].

**Table 3:** Examples of modulators of mitoKv channels, their off-target activity, and clinical use.

Name	Function	Example of Off-Target Activity	Clinical Use	Ref.
Flupirtine	Opener	Activator of Kv7.2/Kv7.3 heterotetramer, Kv7.2, Kv7.3 and Kv7.5, NMDA antagonist, normalizes the level of glutathione, increases the expression of Bcl-2.	Approved for treatment of acute and chronic musculoskeletal pain. Anticonvulsant.	[217,218,220,222]
Retigabine (ezogabine)	Opener	Activator of Kv7.2/Kv7.3 heterotetramer, Kv7.2, Kv7.3 and Kv7.5, modulator of GABAA receptors, inhibitor of Kv2.1.	Potentially effective painkiller, potentially effective in the treatment of mental and neurodegenerative diseases, approved as an anticonvulsant drug in years 2011–2017.	[222,223,224,226,227,228,230,234,237,239,240]
Psora-4	Inhibitor	Inhibitor of plasma membrane Kv1.3, promotes the differentiation and maturation of neurons.	Potentially effective in the treatment of cancer, autoimmune diseases, and multiple sclerosis.	[199,207,208,209]
PAP-1 and PAP-1-MHEG	Inhibitor	Inhibitor of the respiratory chain complex I, induces ROS.	Potentially effective in the treatment of cancer.	[210,211]
Clofazimine	Inhibitor	Inhibitor of the calcineurin/NFAT pathway, inhibitor of mycobacterial electron transfer chain.	Potentially effective in the treatment of cancer and autoimmune diseases, approved for treatment of leprosy and drug-resistant tuberculosis.	[202,204,206,209]

## 5. Mitochondrial Sodium-Activated Potassium Channels

Sodium-activated potassium channels belonging to the Slo2 (K_Na_) family have been identified in the mitochondria of cardiac tissue [241,242,243]. Two genes encode Slo2 channels in mammalian cells: KCNT2, which encodes Slo2.1 (also known as Slick) channels, and KCNT1, which encodes Slo2.2 (Slack) channels [244]. The conductance of the Slack channel was found to be approximately 180 pS, while Slick was found to be approximately 140 pS [244]. Structurally, a part of the pore domain and the transmembrane S6 segment of the Slack channel share similarities with Slo1 channels [245]. Interestingly, Slo2 channels can form calcium-sensitive channels after interaction with BK_Ca_ channels [244]. The activity of both Slo2.1 and Slo2.2 channels is stimulated by sodium ions [244,246,247]. These channels are also activated by several pharmacological compounds, including bithionol, riluzole, loxapine, and niclosamide [244,247]. Slo2 channels are inhibited by compounds such as bepridil [246], clofilium [248], R56865 [246], and quinidine [244,247] (Table 4). It was found that the activation of mitochondrial Slo2 channels by bithionol induced thallium uptake into isolated mouse cardiac mitochondria as well as mitochondria of *C. elegans* [243]. The activity of mitoSlo2 channels is inhibited by bepridil. It has been proposed that these channels play an important role in cardioprotection, similar to mitoBK_Ca_ and mitoK_ATP_ channels [241,242,243,249]. Recently, using the black lipid membrane technique, sodium-activated potassium channels with a conductance of 150 pS were described in brain mitochondria [250]. Sodium ions have been shown to decrease complex I activity and increase complex IV activity in isolated mitochondria. These effects are accompanied by a slight decrease in mitochondrial ROS synthesis and an increase in mitochondrial membrane potential. It was suggested that these effects were related to the activity of the channel. However, no pharmacology specific to Slo2 channels was used in the study [250].

The modulators used to identify and describe mitoSlo2 channels have multiple targets in the cell. For example, bithionol inhibits several enzymes, such as mammalian mitochondrial glutamate dehydrogenase [251,252], human adenylyl cyclase [253], and N-acyl-phosphatidylethanolamine phospholipase D [254]. Bithionol also induces effects at the cellular level. For example, the application of bithionol induced apoptosis in ovarian cancer cells via cell cycle arrest, ROS generation, and inhibition of autotaxin [255,256]. Bithionol has been identified as an apoptosis-inducing photosensitizer for keratinocytes [257]. Its antiseptic and anthelminthic activities have also been studied, e.g., it inhibits the 3-oxoacyl acyl-carrier-protein reductase of *Plasmodium falciparum* and the large tumor antigen of polyomaviruses [258,259,260]. 

Another cardiac mitoSlo2 channel opener, bepridil, is a known calcium channel inhibitor (inhibiting channels such as L-type Ca^2+^ channels) and is used as an antiarrhythmic drug [261]. Additionally, it was shown that bepridil modulates the activity of a few potassium channels. In HEK 293 cells, a low micromolar concentration of bepridil (2–5 μM) inhibited the slow component of cardiac delayed rectifier K^+^ currents composed of KCNQ1/KCNE1 gene products in a concentration-dependent manner [262]. Additionally, bepridil and R56865 inhibited K_ATP_ channels in guinea pig ventricular myocytes, with an estimated IC50 value of 10.5 μM for outward K_ATP_ channel currents at a +60 mV holding potential and 6.6 μM for inward currents recorded at −60 mV [246]. Another study showed that 1–10 μM bepridil activated the mitoK_ATP_ channels of cardiac mitochondria in guinea pigs and induced cardioprotection against ischemia/reperfusion injury [263]. 

Clofilium is another modulator of Slo2 channels. This inhibitor can modulate a broad spectrum of channels, including Slo3, Kv1.5 [264], and TASK-2 channels [265], as well as NMDA receptors [266]. NMDA inhibition was observed after the application of 0.1 μM of clofilium [266]. Clofilium can also induce apoptosis in human promyelocytic leukemia cells [267]. Although clofilium has not been used for mitoSlo2 channel modulation, it was shown to play a role in mitochondrial DNA (mtDNA) maintenance, which was detected by an increase in mtDNA copy number in *C. elegans* and in yeast. A similar effect was observed in POLG-deficient cultured human fibroblasts after the application of 0.5–2.5 μM clofilium [268]. It was concluded that the observed effects were unrelated to the inhibition of potassium channels and that clofilium has a new target in yeast and human cells [268]. Interestingly, the application of clofilium reduced hypoxia-induced neuronal cell death; however, the neuroprotective mechanisms were related to the inhibition of K^+^ efflux [269].

**Table 4:** Examples of mitochondrial sodium-activated potassium channel modulators and their off-target activity.

Name	Function	Example of Off-Target Activity	Clinical Use	Ref.
Bithionol	Opener	Inhibition of mammalian mitochondrial glutamate dehydrogenase, human adenylyl cyclase, and N-acyl-phosphatidylethanolamine phospholipase D. Induces apoptosis of ovarian cancer cells. Apoptosis-inducing photosensitizer for keratinocytes.	Treatment of helminthic infection, inhibits 3-oxoacyl acylcarrier-protein reductase of *Plasmodium falciparum* and the large tumor antigen of polyomaviruses; inhibits host caspases and also reduces the detrimental effects of anthrax lethal toxin, diphtheria toxin, cholera toxin, *Pseudomonas aeruginosa* exotoxin A, Botulinum neurotoxin, ricin, and Zika.	[250,251,252,253,254,255,256,257,258,259]
Bepridil	Inhibitor	Inhibition of calcium channel. Inhibition of K_ATP_ channels in guinea pig ventricular myocytes.	An antiarrhythmic drug.	[261,262,263]
Clofilium	Inhibitor	Modulation of activity of Slo3, Kv1.5, and TASK-2 channels and NMDA receptors. Influences mtDNA maintenance.	Potentially useful for treatment of POLG-related diseases.	[267,269,270]

## 6. Modulators of Mitochondrial TASK Channels

To date, 15 genes encoding a group of two-pore domain K^+^ channels (K2P) have been identified in mammalian cells [159,271]. The synthesized proteins are distinguished by a unique subunit architecture with a molecular weight of approximately 70 kDa [270,272,273]. The group of K2P channels has been divided into 7 functional groups: TREK, TRAAK, TALK, TWIK, TASK, THIK, and TRESK. TREK (K2P 2, K2P 10) and TRAAK (K2P 4) activity is regulated by pressure, temperature, lipid environment, and the presence of volatile anesthetics. TALK (K2P 16, K2P 17) is activated in an alkaline environment or by the presence of NO, and TWIK (K2P 7, K2P 1, K2P 6) has weak rectifying properties. TASK (K2P 15, K2P 3, K2P 9) is inhibited in an acidic environment and activated by volatile anesthetics. THIK (K2P 12, K2P 13) is inhibited by halothane; lastly, TRESK (K2P 18) is activated by calcium ions. The structure of the K2P channel includes four transmembrane (4TM) domains, two re-entry pore loop (P) domains, two selectivity filters (SF), and intracellular amine and carboxyl terminals. K2P also includes a so-called cap structure, which plays a special role in its regulation [270]. Two identical K2P subunits form a single, central selective K^+^ pore, although some K2P subunits may also form heterodimeric complexes in vivo, e.g., TASK-1 and TASK-3 subunits [274,275]. The 4TM domains of the K2P channel family mediate the background potassium currents observed in many cells of bioelectric excitable tissues, such as the heart, brain, muscles, and sensory organs. Interestingly, K2P channels open in the physiological voltage range of the cell membrane and are simultaneously regulated by a number of neurotransmitters and biochemical mediators. It should be noted that, while single functional channels do not have two pores in their structure, each α-subunit has two P domains in its base sequence, hence the name K2P (two-pore domain and not two-porous) channels [270]. The K2P channel family includes the TASK potassium channel subfamily, composed of TASK-1, TASK-3, and TASK-5 [276,277], all of which display K^+^ outwardly rectifying currents that do not depend on membrane voltage. TASK-1 and TASK-3-mediated currents are highly sensitive to extracellular pH [277,278,279]. TASK-5 has no functional expression [280]. The TASK-3 channel (K_2P_9.1, KCNK9, 8q24) was discovered more than 20 years ago in the plasma membrane of mammalian cells. Since then, TASK-3 potassium channels have also been identified in the inner mitochondrial membrane (mitoTASK-3) of melanocytes, melanomas (WM35 and B16F10), and keratinocytes [6,281,282,283]. The mitoTASK-3 channel was detected in the human keratinocyte HaCaT cell line, with a channel conductance of 83 pS at positive voltages and 12 pS at negative voltages in symmetric 150 mM KCl, as measured by the single-channel patch clamp technique. Lidocaine and an acidic pH (<6.2) have been shown to block channel activity. The mitoTASK-3 channel, similar to its plasma membrane version, is also sensitive to an acidic pH [282,283]. Silencing TASK-3 gene expression leads to changes in mitochondrial structure and induces apoptosis in human melanoma cells [284]. It seems particularly interesting that thus far, only the TASK-3 channel from the K2P channel group has been identified in the inner mitochondrial membrane. The apparent lack of other K2P channels in the IMM to date is all the more curious because, as mentioned above, TASK-3 and TASK-1 can form a functional heterodimer of potassium ion transporting channels [274,275]. This may be due to the tissue-specific localization of the TASK-1 and TASK-3 channels. TASK-3-like channels have been located in the mitochondria of the aldosterone-producing layer of the adrenal cortex zona glomerulosa cells [285]. It was shown that knockdown of the TASK-3 gene caused changes in migration and cell survival in gastric cancer, and that the TASK-3 gene could be a potential target for gastric cancer treatment; however, it was not possible to distinguish the role of mitoTASK-3 in addition to the plasma membrane isoform [286]. The role of mitochondrial potassium channels in cancer development was discussed in a recent review [287]. Studies have also shown that overexpression of TASK-3 channels occurs in several types of cancer, such as melanoma, ovarian carcinoma, and breast cancer [281,282,288,289,290,291]. One of the major problems in distinguishing the role of mitochondrial potassium channels from those located in the plasma membrane is their molecular identity or close similarity. Nevertheless, despite the extensive structural similarity of K2P channels, there are differences in the interaction of small molecule compounds among these channels, which may also explain small structural differences as well as why only TASK-3 is located in the IMM [270]. Unfortunately, there are only a few known small-molecule compounds that modulate the activity of the TASK-3 channel [292]. To date, the verified modulators of TASK-3 channels include: the recently described plant-derived compound withaferin A (known in traditional medicine for centuries) [293], an inhibitor based on THPP (5,6,7,8-tetrahydropyrido [4,3-d]pyrimidine) [292] and its recently synthesized derivatives IN-THPP and mitoIN-THPP [294], ruthenium red (which has an IC50 of 0.7 μM) [295], and DR16 and DR16.1, which can penetrate biological membranes [276]. 

Recently, a study by Zúñiga et al. showed that withaferin A, the active biological component of *Withania somnifera* plant extract, has properties that inhibit the activity of the TASK-3 channel on the cell membrane (Table 5) [293]. In light of recent studies, this is an extremely interesting observation, as the expression of the KCNK9 gene for TASK-3 channels is increased in human breast tumors and lung tumors [295] and in 90% of ovarian tumors [288]. Despite the demonstration of a direct effect of withaferin A on the activity of the TASK-3 channel, it should be noted that it is still unclear whether it only affects the channel located in the plasma membrane or also that in the IMM. Withaferin A also acts on cellular pathways, such as cell cycle arrest [296,297], inhibits apoptosis via activated Akt-mediated inhibition of oxidative stress and ROS-dependent mitochondrial dysfunction in human colorectal cancer cells [298,299], and inhibits endoplasmic reticulum stress-associated apoptosis [300]. Low doses of withaferin A are cardioprotective in ischemia/reperfusion events via upregulation of the antiapoptotic mitochondrial pathway in an AMPK-dependent manner [301]. Another group of recently-synthetized inhibitors of TASK-3 channel activity is the 5,6,7,8-tetrahydropyrido [4,3-d]pyrimidine (THPP)-derived compounds IN-THPP and mitoIN-THPP, which differs by the addition of a triphenylphosphonium (TPP) group [294]. Because of its positive charge, the TPP group directs the mitoIN-THPP compound to the IMM. MitoIN-THPP is thus able to inhibit cancer cell migration to a higher extent than IN-THPP, which suggests that mitoTASK-3 is important in cell melanoma cell survival. It should be mentioned that after accumulation in the IMM, mitoIN-THPP is hydrolyzed to IN-THPP-COOH (the active molecule) and TPP-propyl-OH, which is itself unable to trigger apoptosis [292]. Ruthenium red and Ru360, other potential inhibitors of the TASK-3 potassium channel, are only useful for measurements in isolated TASK-3 channels systems because of their broad spectrum of interactions with different proteins in the cell, e.g., ryanodine receptors and mitochondrial calcium uniporter (MCU), respectively [302,303,304,305]. Because ruthenium red and Ru360 are known compounds that disturb intracellular calcium homeostasis, it is difficult to examine the role of TASK-3 and mitoTASK-3 in cellular processes [295,304,306,307,308]. Two novel TASK-3 channel inhibitors (DR16 and DR16.1) were found using virtual screening, and their inhibitory potency was tested using the whole cell patch clamp technique. DR16 had an IC_50_ of 56.8 μM, and DR16.1 had an IC_50_ of 14.2 μM. DR16 and DR16.1 also have inhibitory potency for the TASK-1 channel, with an IC_50_ of 24.7 μM and IC_50_ of 21.21 μM, respectively [276]. It is worth noting that these compounds are not able to distinguish between TASK-1 and TASK-3, nor between those located in the plasma membrane and IMM.

## 7. Pharmacology of Mitochondrial HCN Channels

Hyperpolarization-activated cyclic nucleotide-gated (HCN) channels are nonselective cation channels encoded by four genes, HCN-1,-2,-3, and -4, located on chromosomes 5, 19, 1, and 15, respectively, in humans. HCN channels belong to the superfamily of channels with six transmembrane segments [311]. Functional channels result from the assembly of four subunits, which together form a centralized pore that regulates ion flow across the membrane. Each subunit has several domains: a transmembrane voltage-sensing domain, a transmembrane pore-forming domain, a cytoplasmic C-linker, and a cytoplasmic cyclic nucleotide-binding domain (CNBD), which is critical for the modulation of the channels by cyclic nucleotides [312,313]. HCN channels also have an auxiliary TRIP8b subunit (Rab8b-interacting protein), which acts as an antagonist of cAMP. In the presence of TRIP8b, HCN channels only open at more hyperpolarized potentials [314]. The cryo-EM structures of the human HCN1 channel (hHCN1) have been determined in the absence and in the presence of cAMP, a physiological ligand of HCN channels [184].

HCN channels are mainly expressed throughout the heart and the central nervous system. HCN1 is highly expressed in the neocortex, hippocampus, cerebellar cortex, brainstem, and spinal cord. HCN2 is especially abundant in thalamic and brainstem nuclei. The expression of HCN3 is relatively modest, scattered throughout the brain. HCN4 is expressed in the olfactory bulb and the thalamus. All HCN subunits are expressed in the peripheral nervous system [315]. In the heart, all four isoforms have been detected and were differentially expressed according to the cardiac region. HCN2 and HCN4 are the most abundant isoforms in most mammalian atria and ventricles [316], which also display a minor presence of HCN1. HCN3 is expressed at a low level throughout cardiac regions. There is also evidence for the expression of HCN channels in cells outside of the nervous system and heart, such as the kidneys [317], pancreas [318], and bladder [319]. All HCN isoforms, except HCN3, are present in the retina [320]. HCN channels are localized mainly in the plasma membrane, although they have also been detected in the mitochondria of rat kidneys, human HEK 293 cells [321], and human cardiac tissue [321].

HCN channels are voltage-gated, and, in contrast to most other voltage-gated Kv channels, they are activated by membrane hyperpolarization [322]. HCN channels are permeable to both Na+ and K+ ions but are weakly selective for potassium compared with other voltage-gated Kv channels [323]. Functionally, they differ from each other in terms of activation time. HCN1 channels are the fastest; HCN4 channels are the slowest; and HCN2 and HCN3 are intermediate. In the case of physiological modulators, HCN channels are activated by direct binding of cyclic nucleotides, including cyclic monophosphate nucleotides, such as cAMP, cGMP, and cCMP. There is also evidence that HCN channels are regulated by changes in intracellular pH [155], cholesterol [324], phosphatidylinositol-4,5-bisphosphate [64], and caveolin 3 [33].

Dysfunction of HCN channels has been implicated in heart arrhythmogenic diseases and nervous system diseases, such as pain disorders, epilepsy, and ataxia [325,326,327]. Many pharmacological compounds influencing the activity of HCN channels affect not only the HCN channels of the cell membrane themselves, but also the mitochondrial HCN channels (Table 6). Unfortunately, some of them also have off-targets. One example is the bradycardic drug ZD7288. Experiments on isolated mitochondria showed that ZD7288 inhibits the flow of ions through HCN channels not only in the heart but also in the kidneys [321]. Additionally, ZD7288 was shown to reduce oxygen consumption coupled to ATP synthesis and hyperpolarization of the inner mitochondrial membrane [321]. ZD7288 may also directly block T-type Ca^2+^ channels in mouse spermatogenic cells [328] and the sodium channel NaV1.4 in dorsal root ganglion [329], and can cause a robust depression of mossy fiber basal synaptic transmission [330]. Another HCN drug, ivabradine, also seems to be targeted to mitochondria. After ischemia/reperfusion mitochondrial stimulation, ivabradine reduced mitochondrial ROS formation and increased mitochondrial ATP production [331]. Ivabradine, a member of benzazepines, was the first clinically approved HCN channel blocker and is used to decrease heart rate. Ivabradine binds to the HCN channel pore and blocks HCN channels in pacemaker cells within the sinoatrial node; however, it is not specific to HCN isoforms. More specific to HCN channel isoforms are the modulators zatebradine and EC18 for HCN4 channels, MEL55A for HCN1 and HCN2 channels, and MEL57A for HCN1 channels. Additionally, another HCN channel modulator, gabapentin (an anesthetic, analgesic, and antiepileptic drug), reduces HCN4 channel-mediated currents in neurons [332] and inhibits mitochondrial branched-chain aminotransferase [333], opening mitochondrial ATP-dependent potassium channels and blocking voltage-gated calcium channels in a mouse model [334]. Lamotrigine, an HCN channel agonist (and anticonvulsant drug) may also block T-type Ca^2+^ channels [335].

## 8. Summary and Perspectives

Over the last dozen years, emerging research on potassium channels clearly indicates their involvement in both protective processes and processes influencing cell death. It seems that one of the targets for the regulation of both cellular protection and apoptotic and necrotic processes is the potassium channels found in the inner mitochondrial membrane. A well-documented phenomenon is a protection of heart muscle cells from I/R injury with the use of small-molecule chemical compounds and potassium channel activators of the IMM, e.g., diazoxide and NS1619. Unfortunately, due to the presence of the same types of potassium channels in both the plasma membrane and the IMM, it is extremely difficult to distinguish their participation in both protective and cytotoxic processes. Additionally, the knockdown of the genes responsible for the expression of individual isoforms does not give the desired results, because potassium channels are usually expressed by the same genes, and the difference may result from alternative splicing. Small-molecule chemical compounds modulating the activity of the potassium ion channels in the plasma membrane also modulate the activity of those in the IMM. Although diazoxide acts through an auxiliary subunit, it exhibits the concentration differences necessary to activate a channel in the plasma membrane as well as that in the IMM. In light of recent studies, it seems extremely important to find ways to regulate the expression or activity of the channel only in the IMM, which could protect heart cells in the processes of ischemia/reperfusion or to induce apoptotic processes in cancer cells. Examining the side effects of small-molecule potassium channel modulators may also reveal synergistic pathways that regulate cytoprotection and cellular cytotoxicity, in addition to modulating potassium channels in the IMM.

## Figures and Tables

**Table 5:** Examples of mitochondrial TASK-3 channels modulators and their off-target activity.

Name	Function	Example of Off-Target Activity	Clinical Use	Ref.
Lidocaine	Inhibitor	Inhibition of voltage-dependent Na^+^ and K^+^ channels; Inhibition of the proliferation and metastasis of breast cancer cells by inhibiting the function of TRPM7 channels; reduces the levels of the tumor markers IL-1, TNF-α, and IL-8; inhibition of lung cancer proliferation.	A local anesthetic; used to treat ventricular tachycardia; increases the sensitivity of breast cancer cells to tamoxifen; enhances the toxicity of the cancer drugs mitomycin C, pirarubicin, softening lotion, and cisplatin.	[309,310]
Withaferin A	Inhibitor	Cell cycle arrest, Akt-mediated inhibition; inhibit on endoplasmic reticulum stress.	Used for centuries in traditional Indian medicine; however, there is no good evidence that it is safe or effective for treating any disease.	[292,295,296,297,298,299]
mitoIN-THPP	Inhibitor	Decrease in cellular ATP even when glycolysis was not impaired, up to an extent similar to that triggered by oligomycin, an inhibitor of the FOF1 ATP-synthase; activates the AMP-dependent kinase; induction of massive mitochondrial fragmentation, increase in ROS level, and apoptosis.		[293]
Ruthenium red and Ru360	Inhibitor	Inhibition of ryanodine receptors and mitochondrial calcium uniporter (MCU).		[294,302,303,304,305]
DR16 and DR16.1	Inhibitor	Inhibition of TASK-1.		[276]

**Table 6:** Examples of modulators of mitoHCN channels and their off-target activity.

Name	Function	Example of Alternative Targets	Clinical Use	Ref.
ZD7288	Inhibitor	Inhibitor of T-type Ca^2+^ and NaV1.4 channels. Reduced oxygen consumption coupled to ATP synthesis and hyperpolarization of the IMM causes a depression of mossy fiber basal synaptic transmission.	Bradycardic drug	[320,321,328,329,330]
Gabapentin	Inhibitor	Inhibitor of mitochondrial branched-chain aminotransferase.	An anesthetic, analgesic, and antiepileptic drug; reduces the symptoms of alcohol withdrawal.	[331,332,336]
Lamotrigine	Inhibitor	Blocks T-type Ca^2+^ channels.	Anticonvulsant drug	[335]

## Data Availability

Data sharing not applicable.

## References

[1] Szabo I., Zoratti M., Biasutto L. (2021). Targeting mitochondrial ion channels for cancer therapy. Redox Biol..

[2] Rotko D., Kunz W.S., Szewczyk A., Kulawiak B. (2020). Signaling pathways targeting mitochondrial potassium channels. Int. J. Biochem. Cell. Biol..

[3] Kulawiak B., Bednarczyk P., Szewczyk A. (2021). Multidimensional Regulation of Cardiac Mitochondrial Potassium Channels. Cells.

[4] Szewczyk A., Bednarczyk P., Jedraszko J., Kampa R.P., Koprowski P., Krajewska M., Kucman S., Kulawiak B., Laskowski M., Rotko D. (2018). Mitochondrial potassium channels—An overview. Postepy Biochem..

[5] Urbani A., Prosdocimi E., Carrer A., Checchetto V., Szabo I. (2020). Mitochondrial Ion Channels of the Inner Membrane and Their Regulation in Cell Death Signaling. Front. Cell. Dev. Biol..

[6] Checchetto V., Leanza L., De Stefani D., Rizzuto R., Gulbins E., Szabo I. (2021). Mitochondrial K^+^ channels and their implications for disease mechanisms. Pharmacol. Ther..

[7] Inoue I., Nagase H., Kishi K., Higuti T. (1991). ATP-sensitive K^+^ channel in the mitochondrial inner membrane. Nature.

[8] Paucek P., Mironova G., Mahdi F., Beavis A.D., Woldegiorgis G., Garlid K.D. (1992). Reconstitution and partial purification of the glibenclamide-sensitive, ATP-dependent K+ channel from rat liver and beef heart mitochondria. J. Biol. Chem..

[9] Foster D.B., Ho A.S., Rucker J., Garlid A.O., Chen L., Sidor A., Garlid K.D., O’Rourke B. (2012). Mitochondrial ROMK channel is a molecular component of mitoK_(ATP)_. Circ. Res..

[10] Paggio A., Checchetto V., Campo A., Menabo R., Di Marco G., Di Lisa F., Szabo I., Rizzuto R., De Stefani D. (2019). Identification of an ATP-sensitive potassium channel in mitochondria. Nature.

[11] Choma K., Bednarczyk P., Koszela-Piotrowska I., Kulawiak B., Kudin A., Kunz W.S., Dolowy K., Szewczyk A. (2009). Single channel studies of the ATP-regulated potassium channel in brain mitochondria. J. Bioenergy Biomembr..

[12] Debska G., May R., Kicinska A., Szewczyk A., Elger C.E., Kunz W.S. (2001). Potassium channel openers depolarize hippocampal mitochondria. Brain Res..

[13] Bajgar R., Seetharaman S., Kowaltowski A.J., Garlid K.D., Paucek P. (2001). Identification and properties of a novel intracellular (mitochondrial) ATP-sensitive potassium channel in brain. J. Biol. Chem..

[14] Debska G., Kicinska A., Skalska J., Szewczyk A., May R., Elger C.E., Kunz W.S. (2002). Opening of potassium channels modulates mitochondrial function in rat skeletal muscle. Biochim. Biophys. Acta.

[15] Dahlem Y.A., Horn T.F., Buntinas L., Gonoi T., Wolf G., Siemen D. (2004). The human mitochondrial K_ATP_ channel is modulated by calcium and nitric oxide: A patch-clamp approach. Biochim. Biophys. Acta.

[16] Bednarczyk P., Kicinska A., Laskowski M., Kulawiak B., Kampa R., Walewska A., Krajewska M., Jarmuszkiewicz W., Szewczyk A. (2018). Evidence for a mitochondrial ATP-regulated potassium channel in human dermal fibroblasts. Biochim. Biophys. Acta Bioenergy.

[17] Szabo I., Zoratti M. (2014). Mitochondrial channels: Ion fluxes and more. Physiol. Rev..

[18] Bednarczyk P., Kicinska A., Kominkova V., Ondrias K., Dolowy K., Szewczyk A. (2004). Quinine inhibits mitochondrial ATP-regulated potassium channel from bovine heart. J. Membr. Biol..

[19] Kravenska Y., Checchetto V., Szabo I. (2021). Routes for Potassium Ions across Mitochondrial Membranes: A Biophysical Point of View with Special Focus on the ATP-Sensitive K^+^ Channel. Biomolecules.

[20] Lacza Z., Snipes J.A., Miller A.W., Szabo C., Grover G., Busija D.W. (2003). Heart mitochondria contain functional ATP-dependent K+ channels. J. Mol. Cell. Cardiol..

[21] Laskowski M., Augustynek B., Bednarczyk P., Zochowska M., Kalisz J., O’Rourke B., Szewczyk A., Kulawiak B. (2019). Single-Channel Properties of the ROMK-Pore-Forming Subunit of the Mitochondrial ATP-Sensitive Potassium Channel. Int. J. Mol. Sci..

[22] Juhaszova M., Kobrinsky E., Zorov D.B., Nuss H.B., Yaniv Y., Fishbein K.W., de Cabo R., Montoliu L., Gabelli S.B., Aon M.A. (2019). ATP synthase K^+^-and H^+^-flux drive ATP synthesis and enable mitochondrial K^+^-uniporter function. bioRxiv.

[23] Laskowski M., Augustynek B., Kulawiak B., Koprowski P., Bednarczyk P., Jarmuszkiewicz W., Szewczyk A. (2016). What do we not know about mitochondrial potassium channels?. Biochim. Biophys. Acta.

[24] Testai L., Rapposelli S., Martelli A., Breschi M.C., Calderone V. (2015). Mitochondrial potassium channels as pharmacological target for cardioprotective drugs. Med. Res. Rev..

[25] Pereira O., Kowaltowski A.J. (2021). Mitochondrial K^+^ Transport: Modulation and Functional Consequences. Molecules.

[26] Szewczyk A., Kajma A., Malinska D., Wrzosek A., Bednarczyk P., Zablocka B., Dolowy K. (2010). Pharmacology of mitochondrial potassium channels: Dark side of the field. FEBS Lett..

[27] Szewczyk A., Skalska J., Glab M., Kulawiak B., Malinska D., Koszela-Piotrowska I., Kunz W.S. (2006). Mitochondrial potassium channels: From pharmacology to function. Biochim. Biophys. Acta.

[28] Garlid K.D., Paucek P., Yarov-Yarovoy V., Sun X., Schindler P.A. (1996). The mitochondrial K_ATP_ channel as a receptor for potassium channel openers. J. Biol. Chem..

[29] Busija D.W., Katakam P., Rajapakse N.C., Kis B., Grover G., Domoki F., Bari F. (2005). Effects of ATP-sensitive potassium channel activators diazoxide and BMS-191095 on membrane potential and reactive oxygen species production in isolated piglet mitochondria. Brain Res. Bull..

[30] Kim M.Y., Kim M.J., Yoon I.S., Ahn J.H., Lee S.H., Baik E.J., Moon C.H., Jung Y.S. (2006). Diazoxide acts more as a PKC-e activator, and indirectly activates the mitochondrial K_ATP_ channel conferring cardioprotection against hypoxic injury. Br. J. Pharmacol..

[31] Grover G.J., Atwal K.S. (2002). Pharmacologic profile of the selective mitochondrial-K_ATP_ opener BMS-191095 for treatment of acute myocardial ischemia. Cardiovasc. Drug Rev..

[32] Grover G.J., D’Alonzo A.J., Garlid K.D., Bajgar R., Lodge N.J., Sleph P.G., Darbenzio R.B., Hess T.A., Smith M.A., Paucek P. (2001). Pharmacologic characterization of BMS-191095, a mitochondrial K_ATP_ opener with no peripheral vasodilator or cardiac action potential shortening activity. J. Pharmacol. Exp. Ther..

[33] Bednarczyk P., Dolowy K., Szewczyk A. (2008). New properties of mitochondrial ATP-regulated potassium channels. J. Bioenergy Biomembr..

[34] Ahmad N., Wang Y., Haider K.H., Wang B., Pasha Z., Uzun O., Ashraf M. (2006). Cardiac protection by mitoK_ATP_ channels is dependent on Akt translocation from cytosol to mitochondria during late preconditioning. Am. J. Physiol. Heart Circ. Physiol..

[35] Moses M.A., Addison P.D., Neligan P.C., Ashrafpour H., Huang N., Zair M., Rassuli A., Forrest C.R., Grover G.J., Pang C.Y. (2005). Mitochondrial K_ATP_ channels in hindlimb remote ischemic preconditioning of skeletal muscle against infarction. Am. J. Physiol. Heart Circ. Physiol..

[36] Kis B., Nagy K., Snipes J.A., Rajapakse N.C., Horiguchi T., Grover G.J., Busija D.W. (2004). The mitochondrial K_ATP_ channel opener BMS-191095 induces neuronal preconditioning. Neuroreport.

[37] Katakam P.V., Dutta S., Sure V.N., Grovenburg S.M., Gordon A.O., Peterson N.R., Rutkai I., Busija D.W. (2016). Depolarization of mitochondria in neurons promotes activation of nitric oxide synthase and generation of nitric oxide. Am. J. Physiol. Heart Circ. Physiol..

[38] Mayanagi K., Gaspar T., Katakam P.V., Kis B., Busija D.W. (2007). The mitochondrial K_ATP_ channel opener BMS-191095 reduces neuronal damage after transient focal cerebral ischemia in rats. J. Cereb. Blood Flow Metab..

[39] Gaspar T., Snipes J.A., Busija A.R., Kis B., Domoki F., Bari F., Busija D.W. (2008). ROS-independent preconditioning in neurons via activation of mitoK_ATP_ channels by BMS-191095. J. Cereb. Blood Flow Metab..

[40] Cho M.R., Park J.W., Jung I.S., Yi K.Y., Yoo S.E., Chung H.J., Yun Y.P., Kwon S.H., Shin H.S. (2005). BMS-191095, a cardioselective mitochondrial K_ATP_ opener, inhibits human platelet aggregation by opening mitochondrial K_ATP_ channels. Arch. Pharm. Res..

[41] Malinska D., Kulawiak B., Wrzosek A., Kunz W.S., Szewczyk A. (2010). The cytoprotective action of the potassium channel opener BMS-191095 in C2C12 myoblasts is related to the modulation of calcium homeostasis. Cell Physiol. Biochem..

[42] Sato T., Sasaki N., O’Rourke B., Marbán E. (2000). Nicorandil, a potent cardioprotective agent, acts by opening mitochondrial ATP-dependent potassium channels. J. Am. Coll. Cardiol..

[43] Sanchez-Duarte E., Trujillo X., Cortes-Rojo C., Saavedra-Molina A., Camargo G., Hernandez L., Huerta M., Montoya-Perez R. (2017). Nicorandil improves post-fatigue tension in slow skeletal muscle fibers by modulating glutathione redox state. J. Bioenergy Biomembr..

[44] Tsuchida A., Miura T., Tanno M., Sakamoto J., Miki T., Kuno A., Matsumoto T., Ohnuma Y., Ichikawa Y., Shimamoto K. (2002). Infarct size limitation by nicorandil. J. Am. Coll. Cardiol..

[45] Watanabe Y. (2019). Cardiac Na^+^/Ca^2+^ exchange stimulators among cardioprotective drugs. J. Physiol. Sci..

[46] Taira N. (1989). Nicorandil as a hybrid between nitrates and potassium channel activators. Am. J. Cardiol..

[47] Mano T., Shinohara R., Nagasaka A., Nakagawa H., Uchimura K., Hayashi R., Nakano I., Tsugawa T., Watanabe F., Kobayashi T. (2000). Scavenging effect of nicorandil on free radicals and lipid peroxide in streptozotocin-induced diabetic rats. Metabolism.

[48] Serizawa K., Yogo K., Aizawa K., Tashiro Y., Ishizuka N. (2011). Nicorandil prevents endothelial dysfunction due to antioxidative effects via normalisation of NADPH oxidase and nitric oxide synthase in streptozotocin diabetic rats. Cardiovasc. Diabetol..

[49] Sanchez-Duarte E., Cortes-Rojo C., Sanchez-Briones L.A., Campos-Garcia J., Saavedra-Molina A., Delgado-Enciso I., Lopez-Lemus U.A., Montoya-Perez R. (2020). Nicorandil Affects Mitochondrial Respiratory Chain Function by Increasing Complex III Activity and ROS Production in Skeletal Muscle Mitochondria. J. Membr. Biol..

[50] Afzal M.Z., Reiter M., Gastonguay C., McGivern J.V., Guan X., Ge Z.D., Mack D.L., Childers M.K., Ebert A.D., Strande J.L. (2016). Nicorandil, a Nitric Oxide Donor and ATP-Sensitive Potassium Channel Opener, Protects Against Dystrophin-Deficient Cardiomyopathy. J. Cardiovasc. Pharmacol. Ther..

[51] Chao H.H., Hong H.J., Cheng T.H., Shih N.L., Loh S.H., Liu J.C., Chen J.J., Sung L.C. (2016). Nicorandil Inhibits Cyclic Strain-Induced Interleukin-8 Expression in Human Umbilical Vein Endothelial Cells. Pharmacology.

[52] Wojtovich A.P., Brookes P.S. (2009). The complex II inhibitor atpenin A5 protects against cardiac ischemia-reperfusion injury via activation of mitochondrial K_ATP_ channels. Basic Res. Cardiol..

[53] Wojtovich A.P., Brookes P.S. (2008). The endogenous mitochondrial complex II inhibitor malonate regulates mitochondrial ATP-sensitive potassium channels: Implications for ischemic preconditioning. Biochim. Biophys. Acta.

[54] Li X., Rapedius M., Baukrowitz T., Liu G.X., Srivastava D.K., Daut J., Hanley P.J. (2010). 5-Hydroxydecanoate and coenzyme A are inhibitors of native sarcolemmal KATP channels in inside-out patches. Biochim. Biophys. Acta.

[55] Hanley P.J., Gopalan K.V., Lareau R.A., Srivastava D.K., von Meltzer M., Daut J. (2003). Beta-oxidation of 5-hydroxydecanoate, a putative blocker of mitochondrial ATP-sensitive potassium channels. J. Physiol..

[56] Hanley P.J., Drose S., Brandt U., Lareau R.A., Banerjee A.L., Srivastava D.K., Banaszak L.J., Barycki J.J., Van Veldhoven P.P., Daut J. (2005). 5-Hydroxydecanoate is metabolised in mitochondria and creates a rate-limiting bottleneck for beta-oxidation of fatty acids. J. Physiol..

[57] Drose S., Brandt U., Hanley P.J. (2006). K^+^-independent actions of diazoxide question the role of inner membrane K_ATP_ channels in mitochondrial cytoprotective signaling. J. Biol. Chem..

[58] Ziemys A., Toleikis A., Kopustinskiene D.M. (2006). Molecular modelling of K_ATP_ channel blockers-ADP/ ATP carrier interactions. Syst. Biol..

[59] Skalska J., Debska G., Kunz W.S., Szewczyk A. (2005). Antidiabetic sulphonylureas activate mitochondrial permeability transition in rat skeletal muscle. Br. J. Pharmacol..

[60] Tominaga M., Horie M., Sasayama S., Okada Y. (1995). Glibenclamide, an ATP-sensitive K^+^ channel blocker, inhibits cardiac cAMP-activated Cl^-^ conductance. Circ. Res..

[61] Sakaguchi M., Matsuura H., Ehara T. (1997). Swelling-induced Cl^-^ current in guinea-pig atrial myocytes: Inhibition by glibenclamide. J. Physiol..

[62] Swale D.R., Sheehan J.H., Banerjee S., Husni A.S., Nguyen T.T., Meiler J., Denton J.S. (2015). Computational and functional analyses of a small-molecule binding site in ROMK. Biophys. J..

[63] Weaver C.D., Denton J.S. (2021). Next-generation inward rectifier potassium channel modulators: Discovery and molecular pharmacology. Am. J. Physiol. Cell. Physiol..

[64] Bhave G., Chauder B.A., Liu W., Dawson E.S., Kadakia R., Nguyen T.T., Lewis L.M., Meiler J., Weaver C.D., Satlin L.M. (2011). Development of a selective small-molecule inhibitor of Kir1.1, the renal outer medullary potassium channel. Mol. Pharmacol..

[65] Papanicolaou K.N., Ashok D., Liu T., Bauer T.M., Sun J., Li Z., da Costa E., D’Orleans C.C., Nathan S., Lefer D.J. (2020). Global knockout of ROMK potassium channel worsens cardiac ischemia-reperfusion injury but cardiomyocyte-specific knockout does not: Implications for the identity of mitoKATP. J. Mol. Cell. Cardiol..

[66] Siemen D., Loupatatzis C., Borecky J., Gulbins E., Lang F. (1999). Ca^2+^-activated K channel of the BK-type in the inner mitochondrial membrane of a human glioma cell line. Biochem. Biophys. Res. Commun..

[67] De Marchi U., Sassi N., Fioretti B., Catacuzzeno L., Cereghetti G.M., Szabo I., Zoratti M. (2009). Intermediate conductance Ca^2+^-activated potassium channel (K_Ca_3.1) in the inner mitochondrial membrane of human colon cancer cells. Cell. Calcium.

[68] Dolga A.M., Netter M.F., Perocchi F., Doti N., Meissner L., Tobaben S., Grohm J., Zischka H., Plesnila N., Decher N. (2013). Mitochondrial Small Conductance SK2 Channels Prevent Glutamate-induced Oxytosis and Mitochondrial Dysfunction*. J. Biol. Chem..

[69] Xu W., Liu Y., Wang S., McDonald T., Van Eyk J.E., Sidor A., O’Rourke B. (2002). Cytoprotective role of Ca^2+^- activated K^+^ channels in the cardiac inner mitochondrial membrane. Science.

[70] Douglas R.M., Lai J.C.K., Bian S., Cummins L., Moczydlowski E., Haddad G.G. (2006). The calcium-sensitive large-conductance potassium channel (BK/MAXI K) is present in the inner mitochondrial membrane of rat brain. Neuroscience.

[71] Skalska J., Bednarczyk P., Piwońska M., Kulawiak B., Wilczynski G., Dołowy K., Kunz W., Kudin A., Szewczyk A. (2009). Calcium Ions Regulate K+ Uptake into Brain Mitochondria: The Evidence for a Novel Potassium Channel. Int. J. Mol. Sci..

[72] Skalska J., Piwońska M., Wyroba E., Surmacz L., Wieczorek R., Koszela-Piotrowska I., Zielińska J., Bednarczyk P., Dołowy K., Wilczynski G.M. (2008). A novel potassium channel in skeletal muscle mitochondria. Biochim. Biophys. Acta Bioenergy.

[73] Bednarczyk P., Koziel A., Jarmuszkiewicz W., Szewczyk A. (2013). Large-conductance Ca^2+^-activated potassium channel in mitochondria of endothelial EA.hy926 cells. Am. J. Physiol. Heart Circ. Physiol..

[74] Kicinska A., Augustynek B., Kulawiak B., Jarmuszkiewicz W., Szewczyk A., Bednarczyk P. (2016). A large-conductance calcium-regulated K^+^ channel in human dermal fibroblast mitochondria. Biochem. J..

[75] Shrum S., Rusch N.J., MacMillan-Crow L.A. (2019). Specific BK Channel Activator NS11021 Protects Rat Renal Proximal Tubular Cells from Cold Storage-Induced Mitochondrial Injury In Vitro. Biomolecules.

[76] Sek A., Kampa R.P., Kulawiak B., Szewczyk A., Bednarczyk P. (2021). Identification of the Large-Conductance Ca^2+^-Regulated Potassium Channel in Mitochondria of Human Bronchial Epithelial Cells. Molecules.

[77] Laskowski M., Kicinska A., Szewczyk A., Jarmuszkiewicz W. (2015). Mitochondrial large-conductance potassium channel from *Dictyostelium discoideum*. Int. J. Biochem. Cell. Biol..

[78] Jarmuszkiewicz W., Matkovic K., Koszela-Piotrowska I. (2010). Potassium channels in the mitochondria of unicellular eukaryotes and plants. FEBS Lett..

[79] Matkovic K., Koszela-Piotrowska I., Jarmuszkiewicz W., Szewczyk A. (2011). Ion conductance pathways in potato tuber (*Solanum tuberosum*) inner mitochondrial membrane. Biochim. Biophys. Acta.

[80] Gonzalez-Sanabria N., Echeverria F., Segura I., Alvarado-Sanchez R., Latorre R. (2021). BK in Double-Membrane Organelles: A Biophysical, Pharmacological, and Functional Survey. Front. Physiol..

[81] Sakai Y., Harvey M., Sokolowski B. (2011). Identification and quantification of full-length BK channel variants in the developing mouse cochlea. J. Neurosci. Res..

[82] Singh H., Lu R., Bopassa J.C., Meredith A.L., Stefani E., Toro L. (2013). MitoBK_Ca_ is encoded by the *Kcnma1* gene, and a splicing sequence defines its mitochondrial location. Proc. Natl. Acad. Sci. USA.

[83] Gałecka S., Kulawiak B., Bednarczyk P., Singh H., Szewczyk A. (2021). Single channel properties of mitochondrial large conductance potassium channel formed by BK-VEDEC splice variant. Sci. Rep..

[84] Jaggar J.H., Li A., Parfenova H., Liu J., Umstot E.S., Dopico A.M., Leffler C.W. (2005). Heme is a carbon monoxide receptor for large-conductance Ca^2+^-activated K^+^ channels. Circ. Res..

[85] Tang X.D., Xu R., Reynolds M.F., Garcia M.L., Heinemann S.H., Hoshi T. (2003). Haem can bind to and inhibit mammalian calcium-dependent Slo1 BK channels. Nature.

[86] Augustynek B., Kudin A.P., Bednarczyk P., Szewczyk A., Kunz W.S. (2014). Hemin inhibits the large conductance potassium channel in brain mitochondria: A putative novel mechanism of neurodegeneration. Exp. Neurol..

[87] Brazier S.P., Telezhkin V., Mears R., Muller C.T., Riccardi D., Kemp P.J. (2009). Cysteine residues in the C-terminal tail of the human BK_Ca_a subunit are important for channel sensitivity to carbon monoxide. Adv. Exp. Med. Biol..

[88] Hermann A., Sitdikova G., Weiger T. (2015). Oxidative Stress and Maxi Calcium-Activated Potassium (BK) Channels. Biomolecules.

[89] Rotko D., Bednarczyk P., Koprowski P., Kunz W.S., Szewczyk A., Kulawiak B. (2020). Heme is required for carbon monoxide activation of mitochondrial BK_Ca_ channel. Eur. J. Pharmacol..

[90] Walewska A., Szewczyk A., Koprowski P. (2018). Gas Signaling Molecules and Mitochondrial Potassium Channels. Int. J. Mol. Sci..

[91] Liu Y., Kalogeris T., Wang M., Zuidema M., Wang Q., Dai H., Davis M.J., Hill M.A., Korthuis R.J. (2012). Hydrogen sulfide preconditioning or neutrophil depletion attenuates ischemia-reperfusion-induced mitochondrial dysfunction in rat small intestine. Am. J. Physiol. Gastrointest. Liver Physiol..

[92] Telezhkin V., Brazier S.P., Cayzac S., Muller C.T., Riccardi D., Kemp P.J. (2009). Hydrogen sulfide inhibits human BK_Ca_ channels. Adv. Exp. Med. Biol..

[93] Szteyn K., Singh H. (2020). BK_Ca_ Channels as Targets for Cardioprotection. Antioxidants.

[94] Ohya S., Kuwata Y., Sakamoto K., Muraki K., Imaizumi Y. (2005). Cardioprotective effects of estradiol include the activation of large-conductance Ca^2+^-activated K^+^ channels in cardiac mitochondria. Am. J. Physiol. Heart Circ. Physiol..

[95] Wrzosek A., Augustynek B., Zochowska M., Szewczyk A. (2020). Mitochondrial Potassium Channels as Druggable Targets. Biomolecules.

[96] Debska G., Kicinska A., Dobrucki J., Dworakowska B., Nurowska E., Skalska J., Dolowy K., Szewczyk A. (2003). Large-conductance K^+^ channel openers NS1619 and NS004 as inhibitors of mitochondrial function in glioma cells. Biochem. Pharmacol..

[97] Olesen S.P., Munch E., Moldt P., Drejer J. (1994). Selective activation of Ca^2+^-dependent K^+^ channels by novel benzimidazolone. Eur. J. Pharmacol..

[98] Gribkoff V.K., Champigny G., Barbry P., Dworetzky S.I., Meanwell N.A., Lazdunski M. (1994). The substituted benzimidazolone NS004 is an opener of the cystic fibrosis chloride channel. J. Biol. Chem..

[99] Stowe D.F., Aldakkak M., Camara A.K., Riess M.L., Heinen A., Varadarajan S.G., Jiang M.T. (2006). Cardiac mitochondrial preconditioning by Big Ca^2+^-sensitive K^+^ channel opening requires superoxide radical generation. Am. J. Physiol. Heart Circ. Physiol..

[100] Rockman M.E., Vouga A.G., Rothberg B.S. (2020). Molecular mechanism of BK channel activation by the smooth muscle relaxant NS11021. J. Gen. Physiol..

[101] Gessner G., Cui Y.M., Otani Y., Ohwada T., Soom M., Hoshi T., Heinemann S.H. (2012). Molecular mechanism of pharmacological activation of BK channels. Proc. Natl. Acad. Sci. USA.

[102] Cancherini D.V., Queliconi B.B., Kowaltowski A.J. (2007). Pharmacological and physiological stimuli do not promote Ca^2+^-sensitive K+channel activity in isolated heart mitochondria. Cardiovasc. Res..

[103] Kicinska A., Szewczyk A. (2004). Large-Conductance Potassium Cation Channel Opener NS1619 Inhibits Cardiac Mitochondria Respiratory Chain. Toxicol. Mech. Methods.

[104] Łukasiak A., Skup A., Chlopicki S., Łomnicka M., Kaczara P., Proniewski B., Szewczyk A., Wrzosek A. (2016). SERCA, complex I of the respiratory chain and ATP-synthase inhibition are involved in pleiotropic effects of NS1619 on endothelial cells. Eur. J. Pharmacol..

[105] Park W.S., Kang S.H., Son Y.K., Kim N., Ko J.-H., Kim H.K., Ko E.A., Kim C.D., Han J. (2007). The mitochondrial Ca^2+^-activated K^+^ channel activator, NS 1619 inhibits L-type Ca^2+^ channels in rat ventricular myocytes. Biochem. Biophys. Res. Commun..

[106] Saleh S.N., Angermann J.E., Sones W.R., Leblanc N., Greenwood I.A. (2007). Stimulation of Ca^2+^-gated Cl^-^ currents by the calcium-dependent K^+^ channel modulators NS1619 [1,3-dihydro-1-[2-hydroxy-5-(trifluoromethyl)phenyl]-5-(trifluoromethyl)-2H-benzi midazol-2-one] and isopimaric acid. J. Pharmacol. Exp. Ther..

[107] Gaspar T., Domoki F., Lenti L., Katakam P.V., Snipes J.A., Bari F., Busija D.W. (2009). Immediate neuronal preconditioning by NS1619. Brain Res..

[108] Gaspar T., Katakam P., Snipes J.A., Kis B., Domoki F., Bari F., Busija D.W. (2008). Delayed neuronal preconditioning by NS1619 is independent of calcium activated potassium channels. J. Neurochem..

[109] Bentzen B.H., Nardi A., Calloe K., Madsen L.S., Olesen S.P., Grunnet M. (2007). The small molecule NS11021 is a potent and specific activator of Ca^2+^-activated big-conductance K^+^ channels. Mol. Pharmacol..

[110] Bentzen B.H., Osadchii O., Jespersen T., Hansen R.S., Olesen S.P., Grunnet M. (2009). Activation of big conductance Ca^2+^-activated K^+^ channels (BK) protects the heart against ischemia-reperfusion injury. Pflugers Arch..

[111] Bentzen B.H., Olesen S.P., Ronn L.C., Grunnet M. (2014). BK channel activators and their therapeutic perspectives. Front. Physiol..

[112] Aon M.A., Cortassa S., Wei A.C., Grunnet M., O’Rourke B. (2010). Energetic performance is improved by specific activation of K^+^ fluxes through K_Ca_ channels in heart mitochondria. Biochim. Biophys. Acta.

[113] Augustynek B., Koprowski P., Rotko D., Kunz W., Szewczyk A., Kulawiak B. (2018). Mitochondrial BK Channel Openers CGS7181 and CGS7184 Exhibit Cytotoxic Properties. Int. J. Mol. Sci..

[114] Kulawiak B., Kudin A.P., Szewczyk A., Kunz W.S. (2008). BK channel openers inhibit ROS production of isolated rat brain mitochondria. Exp. Neurol..

[115] Debska-Vielhaber G., Godlewski M.M., Kicinska A., Skalska J., Kulawiak B., Piwonska M., Zablocki K., Kunz W.S., Szewczyk A., Motyl T. (2009). Large-conductance K^+^ channel openers induce death of human glioma cells. J. Physiol. Pharmacol..

[116] Wrzosek A., Tomaskova Z., Ondrias K., Łukasiak A., Szewczyk A. (2012). The potassium channel opener CGS7184 activates Ca^2+^ release from the endoplasmic reticulum. Eur. J. Pharmacol..

[117] Wrzosek A. (2014). The potassium channel opener NS1619 modulates calcium homeostasis in muscle cells by inhibiting SERCA. Cell. Calcium.

[118] Wrzosek A., Tomaskova Z., Ondrias K., Łukasiak A., Szewczyk A. (2012). CGS7184 a potassium channel opener modulates activity of mitochondria and Ca^2+^ homeostasis. Biochim. Biophys. Acta Bioenergy.

[119] Liu Y.-C., Lo Y.-K., Wu S.-N. (2003). Stimulatory effects of chlorzoxazone, a centrally acting muscle relaxant, on large conductance calcium-activated potassium channels in pituitary GH3 cells. Brain Res..

[120] Du X., Carvalho-de-Souza J.L., Wei C., Carrasquel-Ursulaez W., Lorenzo Y., Gonzalez N., Kubota T., Staisch J., Hain T., Petrossian N. (2020). Loss-of-function BK channel mutation causes impaired mitochondria and progressive cerebellar ataxia. Proc. Natl. Acad. Sci. USA.

[121] Sakamoto K., Nonomura T., Ohya S., Muraki K., Ohwada T., Imaizumi Y. (2006). Molecular mechanisms for large conductance Ca^2+^-activated K^+^ channel activation by a novel opener, 12,14-dichlorodehydroabietic acid. J. Pharmacol. Exp. Ther..

[122] Sakamoto K., Ohya S., Muraki K., Imaizumi Y. (2008). A novel opener of large-conductance Ca^2+^-activated K^+^ (BK) channel reduces ischemic injury in rat cardiac myocytes by activating mitochondrial K_Ca_ channel. J. Pharmacol. Sci..

[123] Valverde M.A., Rojas P., Amigo J., Cosmelli D., Orio P., Bahamonde M.I., Mann G.E., Vergara C., Latorre R. (1999). Acute activation of Maxi-K channels (hSlo) by estradiol binding to the b subunit. Science.

[124] Vega-Vela N.E., Osorio D., Avila-Rodriguez M., Gonzalez J., García-Segura L.M., Echeverria V., Barreto G.E. (2017). L-Type Calcium Channels Modulation by Estradiol. Mol. Neurobiol..

[125] Hernández-Aquino E., Muriel P., Muriel P. (2017). Chapter 46—Naringenin and the Liver. Liver Pathophysiology.

[126] Testai L., Da Pozzo E., Piano I., Pistelli L., Gargini C., Breschi M.C., Braca A., Martini C., Martelli A., Calderone V. (2017). The Citrus Flavanone Naringenin Produces Cardioprotective Effects in Hearts from 1 Year Old Rat, through Activation of mitoBK Channels. Front. Pharmacol..

[127] Kampa R.P., Kicinska A., Jarmuszkiewicz W., Pasikowska-Piwko M., Dolegowska B., Debowska R., Szewczyk A., Bednarczyk P. (2019). Naringenin as an opener of mitochondrial potassium channels in dermal fibroblasts. Exp. Dermatol..

[128] Hsu H.-T., Tseng Y.-T., Lo Y.-C., Wu S.-N. (2014). Ability of naringenin, a bioflavonoid, to activate M-type potassium current in motor neuron-like cells and to increase BKCa-channel activity in HEK293T cells transfected with α-hSlo subunit. BMC Neurosci..

[129] Miller C., Moczydlowski E., Latorre R., Phillips M. (1985). Charybdotoxin, a protein inhibitor of single Ca^2+^-activated K^+^ channels from mammalian skeletal muscle. Nature.

[130] Kang J., Huguenard J.R., Prince D.A. (1996). Development of BK channels in neocortical pyramidal neurons. J. Neurophysiol..

[131] Gu X.Q., Pamenter M.E., Siemen D., Sun X., Haddad G.G. (2014). Mitochondrial but not plasmalemmal BK channels are hypoxia-sensitive in human glioma. Glia.

[132] Panyi G., Possani L.D., Rodríguez de la Vega R.C., Gáspár R., Varga Z. (2006). K+ channel blockers: Novel tools to inhibit T cell activation leading to specific immunosuppression. Curr. Pharm. Des..

[133] Galvez A., Gimenez-Gallego G., Reuben J.P., Roy-Contancin L., Feigenbaum P., Kaczorowski G.J., Garcia M.L. (1990). Purification and characterization of a unique, potent, peptidyl probe for the high conductance calcium-activated potassium channel from venom of the scorpion Buthus tamulus. J. Biol. Chem..

[134] Fahanik-babaei J., Eliassi A., Jafari A., Sauve R., Salari S., Saghiri R. (2011). Electro-pharmacological profile of a mitochondrial inner membrane big-potassium channel from rat brain. Biochim. Biophys. Acta Biomembr..

[135] Kaczorowski G.J., Garcia M.L. (2016). Developing Molecular Pharmacology of BK Channels for Therapeutic Benefit. Int. Rev. Neurobiol..

[136] Bergeron Z.L., Bingham J.P. (2012). Scorpion toxins specific for potassium K^+^ channels: A historical overview of peptide bioengineering. Toxins.

[137] Li Q., Yan J. (2016). Modulation of BK Channel Function by Auxiliary Beta and Gamma Subunits. Int. Rev. Neurobiol..

[138] Zhou Y., Lingle C.J. (2014). Paxilline inhibits BK channels by an almost exclusively closed-channel block mechanism. J. Gen. Physiol..

[139] Balderas E., Torres N.S., Rosa-Garrido M., Chaudhuri D., Toro L., Stefani E., Olcese R. (2019). MitoBK_Ca_ channel is functionally associated with its regulatory b1 subunit in cardiac mitochondria. J. Physiol..

[140] Bilmen J.G., Wootton L.L., Michelangeli F. (2002). The mechanism of inhibition of the sarco/endoplasmic reticulum Ca^2+^ ATPase by paxilline. Arch. Biochem. Biophys..

[141] Bilmen J.G., Wootton L.L., Godfrey R.E., Smart O.S., Michelangeli F. (2002). Inhibition of SERCA Ca^2+^ pumps by 2-aminoethoxydiphenyl borate (2-APB). 2-APB reduces both Ca^2+^ binding and phosphoryl transfer from ATP, by interfering with the pathway leading to the Ca^2+^-binding sites. Eur. J. Biochem..

[142] Bednarczyk P., Barker G.D., Halestrap A.P. (2008). Determination of the rate of K^+^ movement through potassium channels in isolated rat heart and liver mitochondria. Biochim. Biophys. Acta.

[143] Kulawiak B., Szewczyk A. (2012). Glutamate-induced cell death in HT22 mouse hippocampal cells is attenuated by paxilline, a BK channel inhibitor. Mitochondrion.

[144] Wulff H., Zhorov B.S. (2008). K+Channel Modulators for the Treatment of Neurological Disorders and Autoimmune Diseases. Chem. Rev..

[145] Coburger I., Yang K., Bernert A., Wiesel E., Sahoo N., Swain S.M., Hoshi T., Schonherr R., Heinemann S.H. (2020). Impact of intracellular hemin on N-type inactivation of voltage-gated K^+^ channels. Pflugers Arch..

[146] Pedarzani P., Stocker M. (2008). Molecular and cellular basis of small- and intermediate-conductance, calcium-activated potassium channel function in the brain. Cell. Mol. Life Sci..

[147] Kshatri A.S., Gonzalez-Hernandez A., Giraldez T. (2018). Physiological Roles and Therapeutic Potential of Ca^2+^ Activated Potassium Channels in the Nervous System. Front. Mol. Neurosci..

[148] Kovalenko I., Glasauer A., Schockel L., Sauter D.R., Ehrmann A., Sohler F., Hagebarth A., Novak I., Christian S. (2016). Identification of K_Ca_3.1 Channel as a Novel Regulator of Oxidative Phosphorylation in a Subset of Pancreatic Carcinoma Cell Lines. PLoS ONE.

[149] Strobaek D., Teuber L., Jorgensen T.D., Ahring P.K., Kjaer K., Hansen R.S., Olesen S.P., Christophersen P., Skaaning-Jensen B. (2004). Activation of human IK and SK Ca^2+^ -activated K^+^ channels by NS309 (6,7-dichloro-1H-indole-2,3-dione 3-oxime). Biochim. Biophys. Acta.

[150] John V.H., Dale T.J., Hollands E.C., Chen M.X., Partington L., Downie D.L., Meadows H.J., Trezise D.J. (2007). Novel 384-Well Population Patch Clamp Electrophysiology Assays for Ca^2+^-Activated K+ Channels. J. Biomol. Screen..

[151] Dolga A.M., De Andrade A., Meissner L., Knaus H.G., Höllerhage M., Christophersen P., Zischka H., Plesnila N., Höglinger G.U., Culmsee C. (2014). Subcellular expression and neuroprotective effects of SK channels in human dopaminergic neurons. Cell Death Dis..

[152] Coleman N., Brown B.M., Olivan-Viguera A., Singh V., Olmstead M.M., Valero M.S., Kohler R., Wulff H. (2014). New positive Ca^2+^-activated K^+^ channel gating modulators with selectivity for K_Ca_3.1. Mol. Pharmacol..

[153] Kim T.Y., Terentyeva R., Roder K.H., Li W., Liu M., Greener I., Hamilton S., Polina I., Murphy K.R., Clements R.T. (2017). SK channel enhancers attenuate Ca^2+^-dependent arrhythmia in hypertrophic hearts by regulating mito-ROS-dependent oxidation and activity of RyR. Cardiovasc. Res..

[154] Kleger A., Seufferlein T., Malan D., Tischendorf M., Storch A., Wolheim A., Latz S., Protze S., Porzner M., Proepper C. (2010). Modulation of Calcium-Activated Potassium Channels Induces Cardiogenesis of Pluripotent Stem Cells and Enrichment of Pacemaker-Like Cells. Circulation.

[155] Augustynek B., Kunz W.S., Szewczyk A. (2017). Guide to the Pharmacology of Mitochondrial Potassium Channels. Handb. Exp. Pharmacol..

[156] Singh S., Syme C.A., Singh A.K., Devor D.C., Bridges R.J. (2001). Benzimidazolone activators of chloride secretion: Potential therapeutics for cystic fibrosis and chronic obstructive pulmonary disease. J. Pharmacol. Exp. Ther..

[157] Cui M., Qin G., Yu K., Bowers M.S., Zhang M. (2014). Targeting the Small- and Intermediate-Conductance Ca^2+^-Activated Potassium Channels: The Drug-Binding Pocket at the Channel/Calmodulin Interface. Neurosignals.

[158] Richter J.M., Schaefer M., Hill K. (2014). Riluzole activates TRPC5 channels independently of PLC activity. Br. J. Pharmacol..

[159] Duprat F., Lesage F., Patel A.J., Fink M., Romey G., Lazdunski M. (2000). The neuroprotective agent riluzole activates the two P domain K(+) channels TREK-1 and TRAAK. Mol. Pharmacol..

[160] Stefani A., Spadoni F., Bernardi G. (1997). Differential Inhibition by Riluzole, Lamotrigine, and Phenytoin of Sodium and Calcium Currents in Cortical Neurons: Implications for Neuroprotective Strategies. Exp. Neurol..

[161] Yokoo H., Shiraishi S., Kobayashi H., Yanagita T., Yamamoto R., Wada A. (1998). Selective inhibition by riluzole of voltage-dependent sodium channels and catecholamine secretion in adrenal chromaffin cells. Naunyn-Schmiedeberg’s Arch. Pharmacol..

[162] Zona C., Siniscalchi A., Mercuri N.B., Bernardi G. (1998). Riluzole interacts with voltage-activated sodium and potassium currents in cultured rat cortical neurons. Neuroscience.

[163] Geevasinga N., Menon P., Ng K., Van Den Bos M., Byth K., Kiernan M.C., Vucic S. (2016). Riluzole exerts transient modulating effects on cortical and axonal hyperexcitability in ALS. Amyotroph. Lateral Scler. Front. Degener..

[164] Bausch A.R., Roy G. (1996). Volume-sensitive chloride channels blocked by neuroprotective drugs in human glial cells (U-138MG). Glia.

[165] Kretschmer B.D., Kratzer U., Schmidt W.J. (1998). Riluzole, a glutamate release inhibitor, and motor behavior. Naunyn-Schmiedeberg’s Arch. Pharmacol..

[166] Azbill R.D., Mu X., Springer J.E. (2000). Riluzole increases high-affinity glutamate uptake in rat spinal cord synaptosomes. Brain Res..

[167] Brugnara C., Armsby C.C., Sakamoto M., Rifai N., Alper S.L., Platt O. (1995). Oral administration of clotrimazole and blockade of human erythrocyte Ca^++^-activated K^+^ channel: The imidazole ring is not required for inhibitory activity. J. Pharmacol. Exp. Ther..

[168] Ishii T.M., Maylie J., Adelman J.P. (1997). Determinants of Apamin and d-Tubocurarine Block in SK Potassium Channels. J. Biol. Chem..

[169] Logsdon N.J., Kang J., Togo J.A., Christian E.P., Aiyar J. (1997). A Novel Gene, hKCa4, Encodes the Calcium-activated Potassium Channel in Human T Lymphocytes. J. Biol. Chem..

[170] Wu S.N., Li H.F., Jan C.R., Shen A.Y. (1999). Inhibition of Ca^2+^-activated K^+^ current by clotrimazole in rat anterior pituitary GH3 cells. Neuropharmacology.

[171] Ghanshani S., Wulff H., Miller M.J., Rohm H., Neben A., Gutman G.A., Cahalan M.D., Chandy K.G. (2000). Up-regulation of the IK_Ca_1 potassium channel during T-cell activation. Molecular mechanism and functional consequences. J. Biol. Chem..

[172] Wulff H., Miller M.J., Hansel W., Grissmer S., Cahalan M.D., Chandy K.G. (2000). Design of a potent and selective inhibitor of the intermediate-conductance Ca^2+^-activated K^+^ channel, IK_Ca_1: A potential immunosuppressant. Proc. Natl. Acad. Sci. USA.

[173] Wulff H., Kolski-Andreaco A., Sankaranarayanan A., Sabatier J.M., Shakkottai V. (2007). Modulators of small- and intermediate-conductance calcium-activated potassium channels and their therapeutic indications. Curr. Med. Chem..

[174] Yi M., Yu P., Lu Q., Geller H.M., Yu Z., Chen H. (2016). K_Ca_3.1 constitutes a pharmacological target for astrogliosis associated with Alzheimer’s disease. Mol. Cell. Neurosci..

[175] Hougaard C., Eriksen B.L., Jorgensen S., Johansen T.H., Dyhring T., Madsen L.S., Strobaek D., Christophersen P. (2007). Selective positive modulation of the SK3 and SK2 subtypes of small conductance Ca^2+^-activated K^+^ channels. Br. J. Pharmacol..

[176] Honrath B., Krabbendam I.E., Culmsee C., Dolga A.M. (2017). Small conductance Ca^2+^-activated K^+^ channels in the plasma membrane, mitochondria and the ER: Pharmacology and implications in neuronal diseases. Neurochem. Int..

[177] Richter M., Nickel C., Apel L., Kaas A., Dodel R., Culmsee C., Dolga A.M. (2015). SK channel activation modulates mitochondrial respiration and attenuates neuronal HT-22 cell damage induced by H_2_O_2_. Neurochem. Int..

[178] Pedarzani P., McCutcheon J.E., Rogge G., Jensen B.S., Christophersen P., Hougaard C., Strøbæk D., Stocker M. (2005). Specific Enhancement of SK Channel Activity Selectively Potentiates the Afterhyperpolarizing Current IAHP and Modulates the Firing Properties of Hippocampal Pyramidal Neurons*. J. Biol. Chem..

[179] Cuthbert A.W. (2001). Assessment of CFTR chloride channel openers in intact normal and cystic fibrosis murine epithelia. Br. J. Pharmacol..

[180] Honrath B., Matschke L., Meyer T., Magerhans L., Perocchi F., Ganjam G.K., Zischka H., Krasel C., Gerding A., Bakker B.M. (2017). SK2 channels regulate mitochondrial respiration and mitochondrial Ca^2+^ uptake. Cell. Death Differ..

[181] Noh T.K., Bang S.H., Lee Y.J., Cho H.I., Jung M.Y., Kim I., Leem C.H., Chang S.E. (2019). The ion channel activator CyPPA inhibits melanogenesis via the GSK3β/β-catenin pathway. Chem.-Biol. Interact..

[182] Stowe D.F., Gadicherla A.K., Zhou Y., Aldakkak M., Cheng Q., Kwok W.M., Jiang M.T., Heisner J.S., Yang M., Camara A.K. (2013). Protection against cardiac injury by small Ca^2+^-sensitive K^+^ channels identified in guinea pig cardiac inner mitochondrial membrane. Biochim. Biophys. Acta.

[183] Gu H., Han S.M., Park K.-K. (2020). Therapeutic Effects of Apamin as a Bee Venom Component for Non-Neoplastic Disease. Toxins.

[184] Kim W.-H., An H.-J., Kim J.-Y., Gwon M.-G., Gu H., Lee S.-J., Park J.Y., Park K.-D., Han S.-M., Kim M.-K. (2017). Apamin inhibits TNF-α- and IFN-γ-induced inflammatory cytokines and chemokines via suppressions of NF-κB signaling pathway and STAT in human keratinocytes. Pharmacol. Rep..

[185] Strobaek D., Hougaard C., Johansen T.H., Sorensen U.S., Nielsen E.O., Nielsen K.S., Taylor R.D., Pedarzani P., Christophersen P. (2006). Inhibitory gating modulation of small conductance Ca^2+^-activated K^+^ channels by the synthetic compound (R)-N-(benzimidazol-2-yl)-1,2,3,4-tetrahydro-1-naphtylamine (NS8593) reduces afterhyperpolarizing current in hippocampal CA1 neurons. Mol. Pharmacol..

[186] Sorensen U.S., Strobaek D., Christophersen P., Hougaard C., Jensen M.L., Nielsen E.O., Peters D., Teuber L. (2008). Synthesis and structure-activity relationship studies of 2-(N-substituted)-aminobenzimidazoles as potent negative gating modulators ofsmall conductance Ca^2+^-activated K^+^ channels. J. Med. Chem..

[187] Syme C.A., Gerlach A.C., Singh A.K., Devor D.C. (2000). Pharmacological activation of cloned intermediate- and small-conductance Ca^2+^-activated K^+^ channels. Am. J. Physiol. Cell Physiol..

[188] Cao Y., Dreixler J.C., Roizen J.D., Roberts M.T., Houamed K.M. (2001). Modulation of recombinant small-conductance Ca^2+^-activated K^+^ channels by the muscle relaxant chlorzoxazone and structurally related compounds. J. Pharmacol. Exp. Ther..

[189] Alvina K., Khodakhah K. (2010). K_Ca_ channels as therapeutic targets in episodic ataxia type-2. J. Neurosci..

[190] Teisseyre A., Palko-Labuz A., Sroda-Pomianek K., Michalak K. (2019). Voltage-Gated Potassium Channel Kv1.3 as a Target in Therapy of Cancer. Front. Oncol..

[191] Kuang Q., Purhonen P., Hebert H. (2015). Structure of potassium channels. Cell. Mol. Life Sci..

[192] Barros F., de la Pena P., Dominguez P., Sierra L.M., Pardo L.A. (2020). The EAG Voltage-Dependent K^+^ Channel Subfamily: Similarities and Differences in.n Structural Organization and Gating. Front. Pharmacol..

[193] Leanza L., Checchetto V., Biasutto L., Rossa A., Costa R., Bachmann M., Zoratti M., Szabo I. (2019). Pharmacological modulation of mitochondrial ion channels. Br. J. Pharmacol..

[194] Szabo I., Bock J., Jekle A., Soddemann M., Adams C., Lang F., Zoratti M., Gulbins E. (2005). A novel potassium channel in lymphocyte mitochondria. J. Biol. Chem..

[195] Gulbins E., Sassi N., Grassme H., Zoratti M., Szabo I. (2010). Role of Kv1.3 mitochondrial potassium channel in apoptotic signalling in lymphocytes. Biochim. Biophys. Acta.

[196] Jang S.H., Byun J.K., Jeon W.I., Choi S.Y., Park J., Lee B.H., Yang J.E., Park J.B., O’Grady S.M., Kim D.Y. (2015). Nuclear localization and functional characteristics of voltage-gated potassium channel Kv1.3. J. Biol. Chem..

[197] Leanza L., Henry B., Sassi N., Zoratti M., Chandy K.G., Gulbins E., Szabo I. (2012). Inhibitors of mitochondrial Kv1.3 channels induce Bax/Bak-independent death of cancer cells. EMBO Mol. Med..

[198] Comes N., Bielanska J., Vallejo-Gracia A., Serrano-Albarras A., Marruecos L., Gomez D., Soler C., Condom E., Ramon Y.C.S., Hernandez-Losa J. (2013). The voltage-dependent K^+^ channels Kv1.3 and Kv1.5 in human cancer. Front. Physiol..

[199] Leanza L., Trentin L., Becker K.A., Frezzato F., Zoratti M., Semenzato G., Gulbins E., Szabo I. (2013). Clofazimine, Psora-4 and PAP-1, inhibitors of the potassium channel Kv1.3, as a new and selective therapeutic strategy in chronic lymphocytic leukemia. Leukemia.

[200] Szabo I., Bock J., Grassme H., Soddemann M., Wilker B., Lang F., Zoratti M., Gulbins E. (2008). Mitochondrial potassium channel Kv1.3 mediates Bax-induced apoptosis in lymphocytes. Proc. Natl. Acad. Sci. USA.

[201] Zaccagnino A., Manago A., Leanza L., Gontarewitz A., Linder B., Azzolini M., Biasutto L., Zoratti M., Peruzzo R., Legler K. (2017). Tumor-reducing effect of the clinically used drug clofazimine in a SCID mouse model of pancreatic ductal adenocarcinoma. Oncotarget.

[202] Ahmed K., Koval A., Xu J., Bodmer A., Katanaev V.L. (2019). Towards the first targeted therapy for triple-negative breast cancer: Repositioning of clofazimine as a chemotherapy-compatible selective Wnt pathway inhibitor. Cancer Lett..

[203] Ren Y.R., Pan F., Parvez S., Fleig A., Chong C.R., Xu J., Dang Y., Zhang J., Jiang H., Penner R. (2008). Clofazimine inhibits human Kv1.3 potassium channel by perturbing calcium oscillation in T lymphocytes. PLoS ONE.

[204] Faouzi M., Starkus J., Penner R. (2015). State-dependent blocking mechanism of Kv 1.3 channels by the antimycobacterial drug clofazimine. Br. J. Pharmacol..

[205] Love M.S., Beasley F.C., Jumani R.S., Wright T.M., Chatterjee A.K., Huston C.D., Schultz P.G., McNamara C.W. (2017). A high-throughput phenotypic screen identifies clofazimine as a potential treatment for cryptosporidiosis. PLoS Negl. Trop Dis..

[206] Lechartier B., Cole S.T. (2015). Mode of Action of Clofazimine and Combination Therapy with Benzothiazinones against Mycobacterium tuberculosis. Antimicrob. Agents Chemother..

[207] Marzian S., Stansfeld P.J., Rapedius M., Rinne S., Nematian-Ardestani E., Abbruzzese J.L., Steinmeyer K., Sansom M.S., Sanguinetti M.C., Baukrowitz T. (2013). Side pockets provide the basis for a new mechanism of Kv channel-specific inhibition. Nat. Chem. Biol..

[208] Zhou Y.Y., Hou G.Q., He S.W., Xiao Z., Xu H.J., Qiu Y.T., Jiang S., Zheng H., Li Z.Y. (2015). Psora-4, a Kv1.3 Blocker, Enhances Differentiation and Maturation in Neural Progenitor Cells. CNS Neurosci. Ther..

[209] Hyodo T., Oda T., Kikuchi Y., Higashi K., Kushiyama T., Yamamoto K., Yamada M., Suzuki S., Hokari R., Kinoshita M. (2010). Voltage-gated potassium channel Kv1.3 blocker as a potential treatment for rat anti-glomerular basement membrane glomerulonephritis. Am. J. Physiol. Renal Physiol..

[210] Peruzzo R., Mattarei A., Azzolini M., Becker-Flegler K.A., Romio M., Rigoni G., Carrer A., Biasutto L., Parrasia S., Kadow S. (2020). Insight into the mechanism of cytotoxicity of membrane-permeant psoralenic Kv1.3 channel inhibitors by chemical dissection of a novel member of the family. Redox Biol..

[211] Venturini E., Leanza L., Azzolini M., Kadow S., Mattarei A., Weller M., Tabatabai G., Edwards M.J., Zoratti M., Paradisi C. (2017). Targeting the Potassium Channel Kv1.3 Kills Glioblastoma Cells. Neurosignals.

[212] Ostacolo C., Miceli F., Di Sarno V., Nappi P., Iraci N., Soldovieri M.V., Ciaglia T., Ambrosino P., Vestuto V., Lauritano A. (2020). Synthesis and Pharmacological Characterization of Conformationally Restricted Retigabine Analogues as Novel Neuronal Kv7 Channel Activators. J. Med. Chem..

[213] Brickel N., Gandhi P., VanLandingham K., Hammond J., DeRossett S. (2012). The urinary safety profile and secondary renal effects of retigabine (ezogabine): A first-in-class antiepileptic drug that targets KCNQ (K(v)7) potassium channels. Epilepsia.

[214] Testai L., Barrese V., Soldovieri M.V., Ambrosino P., Martelli A., Vinciguerra I., Miceli F., Greenwood I.A., Curtis M.J., Breschi M.C. (2016). Expression and function of Kv7.4 channels in rat cardiac mitochondria: Possible targets for cardioprotection. Cardiovasc. Res..

[215] Devulder J. (2010). Flupirtine in pain management: Pharmacological properties and clinical use. CNS Drugs.

[216] Ipavec V., Martire M., Barrese V., Taglialatela M., Curro D. (2011). KV7 channels regulate muscle tone and nonadrenergic noncholinergic relaxation of the rat gastric fundus. Pharmacol. Res..

[217] Sampath D., Valdez R., White A.M., Raol Y.H. (2017). Anticonvulsant effect of flupirtine in an animal model of neonatal hypoxic-ischemic encephalopathy. Neuropharmacology.

[218] Raffa R.B., Pergolizzi J.V. (2012). The evolving understanding of the analgesic mechanism of action of flupirtine. J. Clin. Pharm. Ther..

[219] Schroder H.C., Muller W.E. (2002). Neuroprotective effect of flupirtine in prion disease. Drugs Today.

[220] Michel M.C., Radziszewski P., Falconer C., Marschall-Kehrel D., Blot K. (2012). Unexpected frequent hepatotoxicity of a prescription drug, flupirtine, marketed for about 30 years. Br. J. Clin. Pharmacol..

[221] Siegmund W., Modess C., Scheuch E., Methling K., Keiser M., Nassif A., Rosskopf D., Bednarski P.J., Borlak J., Terhaag B. (2015). Metabolic activation and analgesic effect of flupirtine in healthy subjects, influence of the polymorphic NAT2, UGT1A1 and GSTP1. Br. J. Clin. Pharmacol..

[222] Porter R.J., Nohria V., Rundfeldt C. (2007). Retigabine. Neurotherapeutics.

[223] Stafstrom C.E., Grippon S., Kirkpatrick P. (2011). Ezogabine (retigabine). Nat. Rev. Drug Discov..

[224] Barrese V., Miceli F., Soldovieri M.V., Ambrosino P., Iannotti F.A., Cilio M.R., Taglialatela M. (2010). Neuronal potassium channel openers in the management of epilepsy: Role and potential of retigabine. Clin. Pharmacol..

[225] Zhang F., Liu S., Jin L., Tang L., Zhao X., Yang T., Wang Y., Huo B., Liu R., Li H. (2020). Antinociceptive Efficacy of Retigabine and Flupirtine for Gout Arthritis Pain. Pharmacology.

[226] Zheng Q., Fang D., Liu M., Cai J., Wan Y., Han J.S., Xing G.G. (2013). Suppression of KCNQ/M (Kv7) potassium channels in dorsal root ganglion neurons contributes to the development of bone cancer pain in a rat model. Pain.

[227] Hayashi H., Iwata M., Tsuchimori N., Matsumoto T. (2014). Activation of peripheral KCNQ channels attenuates inflammatory pain. Mol. Pain.

[228] Li H., Wang F., Wang X., Sun R., Chen J., Chen B., Zhang Y. (2015). Antinociceptive Efficacy of Retigabine in the Monosodium Lodoacetate Rat Model for Osteoarthritis Pain. Pharmacology.

[229] Hirano K., Kuratani K., Fujiyoshi M., Tashiro N., Hayashi E., Kinoshita M. (2007). Kv7.2-7.5 voltage-gated potassium channel (KCNQ2-5) opener, retigabine, reduces capsaicin-induced visceral pain in mice. Neurosci. Lett..

[230] Gunthorpe M.J., Large C.H., Sankar R. (2012). The mechanism of action of retigabine (ezogabine), a first-in-class K^+^ channel opener for the treatment of epilepsy. Epilepsia.

[231] Sotty F., Damgaard T., Montezinho L.P., Mork A., Olsen C.K., Bundgaard C., Husum H. (2009). Antipsychotic-like effect of retigabine [N-(2-Amino-4-(fluorobenzylamino)-phenyl)carbamic acid ester], a KCNQ potassium channel opener, via modulation of mesolimbic dopaminergic neurotransmission. J. Pharmacol. Exp. Ther..

[232] Brodie M.S., Appel S.B. (2000). Dopaminergic neurons in the ventral tegmental area of C57BL/6J and DBA/2J mice differ in sensitivity to ethanol excitation. Alcohol. Clin. Exp. Res..

[233] Zwierzynska E., Krupa A., Pietrzak B. (2015). A pharmaco-EEG study of the interaction between ethanol and retigabine in rabbits. Am. J. Drug Alcohol. Abuse.

[234] Boscia F., Annunziato L., Taglialatela M. (2006). Retigabine and flupirtine exert neuroprotective actions in organotypic hippocampal cultures. Neuropharmacology.

[235] Zwierzynska E., Krupa-Burtnik A., Pietrzak B. (2021). Beneficial effect of retigabine on memory in rats receiving ethanol. Pharmacol. Rep..

[236] Young M.B., Thomas S.A. (2014). M1-muscarinic receptors promote fear memory consolidation via phospholipase C and the M-current. J. Neurosci..

[237] Dencker D., Husum H. (2010). Antimanic efficacy of retigabine in a proposed mouse model of bipolar disorder. Behav. Brain Res..

[238] Dupont C., Denman K.S., Hawash A.A., Voss A.A., Rich M.M. (2019). Treatment of myotonia congenita with retigabine in mice. Exp. Neurol..

[239] Zhao Y.J., Nai Y., Li S.Y., Zheng Y.H. (2018). Retigabine protects the blood-brain barrier by regulating tight junctions between cerebral vascular endothelial cells in cerebral ischemia-reperfusion rats. Eur. Rev. Med. Pharmacol. Sci..

[240] Stas J.I., Bocksteins E., Jensen C.S., Schmitt N., Snyders D.J. (2016). The anticonvulsant retigabine suppresses neuronal KV2-mediated currents. Sci. Rep..

[241] Wojtovich A.P., Smith C.O., Urciuoli W.R., Wang Y.T., Xia X.M., Brookes P.S., Nehrke K. (2016). Cardiac Slo2.1 Is Required for Volatile Anesthetic Stimulation of K+ Transport and Anesthetic Preconditioning. Anesthesiology.

[242] Smith C.O., Wang Y.T., Nadtochiy S.M., Miller J.H., Jonas E.A., Dirksen R.T., Nehrke K., Brookes P.S. (2018). Cardiac metabolic effects of KNa1.2 channel deletion and evidence for its mitochondrial localization. FASEB J..

[243] Wojtovich A.P., Sherman T.A., Nadtochiy S.M., Urciuoli W.R., Brookes P.S., Nehrke K. (2011). SLO-2 is cytoprotective and contributes to mitochondrial potassium transport. PLoS ONE.

[244] Kaczmarek L.K. (2013). Slack, Slick and Sodium-Activated Potassium Channels. ISRN Neurosci..

[245] Joiner W.J., Tang M.D., Wang L.Y., Dworetzky S.I., Boissard C.G., Gan L., Gribkoff V.K., Kaczmarek L.K. (1998). Formation of intermediate-conductance calcium-activated potassium channels by interaction of Slack and Slo subunits. Nat. Neurosci..

[246] Li Y., Sato T., Arita M. (1999). Bepridil blunts the shortening of action potential duration caused by metabolic inhibition via blockade of ATP-sensitive K^+^ channels and Na^+^-activated K^+^ channels. J. Pharmacol. Exp. Ther..

[247] Yang B., Gribkoff V.K., Pan J., Damagnez V., Dworetzky S.I., Boissard C.G., Bhattacharjee A., Yan Y., Sigworth F.J., Kaczmarek L.K. (2006). Pharmacological activation and inhibition of Slack (Slo2.2) channels. Neuropharmacology.

[248] de Los Angeles Tejada M., Stolpe K., Meinild A.K., Klaerke D.A. (2012). Clofilium inhibits Slick and Slack potassium channels. Biologics.

[249] Smith C.O., Nehrke K., Brookes P.S. (2017). The Slo(w) path to identifying the mitochondrial channels responsible for ischemic protection. Biochem. J..

[250] Fahanik-Babaei J., Rezaee B., Nazari M., Torabi N., Saghiri R., Sauve R., Eliassi A. (2020). A new brain mitochondrial sodium-sensitive potassium channel: Effect of sodium ions on respiratory chain activity. J. Cell. Sci..

[251] Li M., Smith C.J., Walker M.T., Smith T.J. (2009). Novel inhibitors complexed with glutamate dehydrogenase: Allosteric regulation by control of protein dynamics. J. Biol. Chem..

[252] Whitelaw B.S., Robinson M.B. (2013). Inhibitors of glutamate dehydrogenase block sodium-dependent glutamate uptake in rat brain membranes. Front. Endocrinol..

[253] Kleinboelting S., Ramos-Espiritu L., Buck H., Colis L., van den Heuvel J., Glickman J.F., Levin L.R., Buck J., Steegborn C. (2016). Bithionol Potently Inhibits Human Soluble Adenylyl Cyclase through Binding to the Allosteric Activator Site. J. Biol. Chem..

[254] Aggarwal G., Zarrow J.E., Mashhadi Z., Flynn C.R., Vinson P., Weaver C.D., Davies S.S. (2020). Symmetrically substituted dichlorophenes inhibit N-acyl-phosphatidylethanolamine phospholipase D. J. Biol. Chem..

[255] Ayyagari V.N., Brard L. (2014). Bithionol inhibits ovarian cancer cell growth in vitro—Studies on mechanism(s) of action. BMC Cancer.

[256] Ayyagari V.N., Hsieh T.H.J., Diaz-Sylvester P.L., Brard L. (2017). Evaluation of the cytotoxicity of the Bithionol—Cisplatin combination in a panel of human ovarian cancer cell lines. BMC Cancer.

[257] Kurita M., Shimauchi T., Kobayashi M., Atarashi K., Mori K., Tokura Y. (2007). Induction of keratinocyte apoptosis by photosensitizing chemicals plus UVA. J. Dermatol. Sci..

[258] Wickramasinghe S.R., Inglis K.A., Urch J.E., Muller S., van Aalten D.M., Fairlamb A.H. (2006). Kinetic, inhibition and structural studies on 3-oxoacyl-ACP reductase from Plasmodium falciparum, a key enzyme in fatty acid biosynthesis. Biochem. J..

[259] Seguin S.P., Ireland A.W., Gupta T., Wright C.M., Miyata Y., Wipf P., Pipas J.M., Gestwicki J.E., Brodsky J.L. (2012). A screen for modulators of large T antigen’s ATPase activity uncovers novel inhibitors of Simian Virus 40 and BK virus replication. Antivir. Res..

[260] Leonardi W., Zilbermintz L., Cheng L.W., Zozaya J., Tran S.H., Elliott J.H., Polukhina K., Manasherob R., Li A., Chi X. (2016). Bithionol blocks pathogenicity of bacterial toxins, ricin, and Zika virus. Sci. Rep..

[261] Hollingshead L.M., Faulds D., Fitton A. (1992). Bepridil. A review of its pharmacological properties and therapeutic use in stable angina pectoris. Drugs.

[262] Yumoto Y., Horie M., Kubota T., Ninomiya T., Kobori A., Takenaka K., Takano M., Niwano S., Izumi T. (2004). Bepridil block of recombinant human cardiac IKs current shows a time-dependent unblock. J. Cardiovasc. Pharmacol..

[263] Sato T., Costa A.D., Saito T., Ogura T., Ishida H., Garlid K.D., Nakaya H. (2006). Bepridil, an antiarrhythmic drug, opens mitochondrial K_ATP_ channels, blocks sarcolemmal K_ATP_ channels, and confers cardioprotection. J. Pharmacol. Exp. Ther..

[264] Malayev A.A., Nelson D.J., Philipson L.H. (1995). Mechanism of clofilium block of the human Kv1.5 delayed rectifier potassium channel. Mol. Pharmacol..

[265] Kirkegaard S.S., Lambert I.H., Gammeltoft S., Hoffmann E.K. (2010). Activation of the TASK-2 channel after cell swelling is dependent on tyrosine phosphorylation. Am. J. Physiol. Cell Physiol..

[266] Yang A., Wang X.Q., Sun C.S., Wei L., Yu S.P. (2005). Inhibitory effects of clofilium on membrane currents associated with Ca channels, NMDA receptor channels and Na+, K+-ATPase in cortical neurons. Pharmacology.

[267] Choi B.Y., Kim H.Y., Lee K.H., Cho Y.H., Kong G. (1999). Clofilium, a potassium channel blocker, induces apoptosis of human promyelocytic leukemia (HL-60) cells via Bcl-2-insensitive activation of caspase-3. Cancer Lett..

[268] Pitayu L., Baruffini E., Rodier C., Rotig A., Lodi T., Delahodde A. (2016). Combined use of *Saccharomyces cerevisiae*, *Caenorhabditis elegans* and patient fibroblasts leads to the identification of clofilium tosylate as a potential therapeutic chemical against POLG-related diseases. Hum. Mol. Genet..

[269] Wei L., Yu S.P., Gottron F., Snider B.J., Zipfel G.J., Choi D.W. (2003). Potassium channel blockers attenuate hypoxia- and ischemia-induced neuronal death in vitro and in vivo. Stroke.

[270] Natale A.M., Deal P.E., Minor D.L. (2021). Structural Insights into the Mechanisms and Pharmacology of K2P Potassium Channels. J. Mol. Biol..

[271] Alexander S., Mathie A., Peters J., Veale E., Striessnig J., Kelly E., Armstrong J., Faccenda E., Harding S., Pawson A. (2019). THE CONCISE GUIDE TO PHARMACOLOGY 2019/20: Ion channels. Br. J. Pharmacol..

[272] Goldstein S.A., Bockenhauer D., O’Kelly I., Zilberberg N. (2001). Potassium leak channels and the KCNK family of two-P-domain subunits. Nat. Rev. Neurosci..

[273] Herrera-Pérez S., Campos-Ríos A., Rueda-Ruzafa L., Lamas J.A. (2021). Contribution of K2P Potassium Channels to Cardiac Physiology and Pathophysiology. Int. J. Mol. Sci..

[274] Czirják G., Enyedi P. (2002). Formation of functional heterodimers between the TASK-1 and TASK-3 two-pore domain potassium channel subunits. J. Biol. Chem..

[275] Lauritzen I., Zanzouri M., Honoré E., Duprat F., Ehrengruber M.U., Lazdunski M., Patel A.J. (2003). K+-dependent cerebellar granule neuron apoptosis. Role of task leak K+ channels. J. Biol. Chem..

[276] Ramírez D., Concha G., Arévalo B., Prent-Peñaloza L., Zúñiga L., Kiper A.K., Rinné S., Reyes-Parada M., Decher N., González W. (2019). Discovery of Novel TASK-3 Channel Blockers Using a Pharmacophore-Based Virtual Screening. Int. J. Mol. Sci..

[277] Enyedi P., Czirják G. (2010). Molecular Background of Leak K+ Currents: Two-Pore Domain Potassium Channels. Physiol. Rev..

[278] Duprat F., Lesage F., Fink M., Reyes R., Heurteaux C., Lazdunski M. (1997). TASK, a human background K+ channel to sense external pH variations near physiological pH. EMBO J..

[279] Rajan S., Wischmeyer E., Xin Liu G., Preisig-Müller R., Daut J., Karschin A., Derst C. (2000). TASK-3, a novel tandem pore domain acid-sensitive K+ channel. An extracellular histiding as pH sensor. J. Biol. Chem..

[280] Kim D., Gnatenco C. (2001). TASK-5, a New Member of the Tandem-Pore K+ Channel Family. Biochem. Biophys. Res. Communications.

[281] Pocsai K., Kosztka L., Bakondi G., Gonczi M., Fodor J., Dienes B., Szentesi P., Kovacs I., Feniger-Barish R., Kopf E. (2006). Melanoma cells exhibit strong intracellular TASK-3-specific immunopositivity in both tissue sections and cell culture. Cell. Mol. Life Sci..

[282] Rusznák Z., Bakondi G., Kosztka L., Pocsai K., Dienes B., Fodor J., Telek A., Gönczi M., Szűcs G., Csernoch L. (2008). Mitochondrial expression of the two-pore domain TASK-3 channels in malignantly transformed and non-malignant human cells. Virchows Archiv.

[283] Toczylowska-Maminska R., Olszewska A., Laskowski M., Bednarczyk P., Skowronek K., Szewczyk A. (2014). Potassium channel in the mitochondria of human keratinocytes. J. Investig. Dermatol..

[284] Nagy D., Gönczi M., Dienes B., Szöőr Á., Fodor J., Nagy Z., Tóth A., Fodor T., Bai P., Szücs G. (2014). Silencing the KCNK9 potassium channel (TASK-3) gene disturbs mitochondrial function, causes mitochondrial depolarization, and induces apoptosis of human melanoma cells. Arch. Dermatol. Res..

[285] Yao J., McHedlishvili D., McIntire W.E., Guagliardo N.A., Erisir A., Coburn C.A., Santarelli V.P., Bayliss D.A., Barrett P.Q. (2017). Functional TASK-3-Like Channels in Mitochondria of Aldosterone-Producing Zona Glomerulosa Cells. Hypertension.

[286] Cikutović-Molina R., Herrada A.A., González W., Brown N., Zúñiga L. (2019). TASK-3 Gene Knockdown Dampens Invasion and Migration and Promotes Apoptosis in KATO III and MKN-45 Human Gastric Adenocarcinoma Cell Lines. Int. J. Mol. Sci..

[287] Bachmann M., Pontarin G., Szabo I. (2019). The Contribution of Mitochondrial Ion Channels to Cancer Development and Progression. Cell. Physiol. Biochem..

[288] Innamaa A., Jackson L., Asher V., Van Shalkwyk G., Warren A., Hay D., Bali A., Sowter H., Khan R. (2013). Expression and prognostic significance of the oncogenic K2P potassium channel KCNK9 (TASK-3) in ovarian carcinoma. Anticancer Res..

[289] Patel A.J., Lazdunski M. (2004). The 2P-domain K+ channels: Role in apoptosis and tumorigenesis. Pflugers Arch..

[290] Mu D., Chen L., Zhang X., See L.-H., Koch C.M., Yen C., Tong J.J., Spiegel L., Nguyen K.C.Q., Servoss A. (2003). Genomic amplification and oncogenic properties of the KCNK9 potassium channel gene. Cancer Cell.

[291] Zúñiga R., Valenzuela C., Concha G., Brown N., Zúñiga L. (2018). TASK-3 Downregulation Triggers Cellular Senescence and Growth Inhibition in Breast Cancer Cell Lines. Int. J. Mol. Sci..

[292] Coburn C.A., Luo Y., Cui M., Wang J., Soll R., Dong J., Hu B., Lyon M.A., Santarelli V.P., Kraus R.L. (2012). Discovery of a pharmacologically active antagonist of the two-pore-domain potassium channel K2P9.1 (TASK-3). ChemMedChem.

[293] Zúñiga R., Concha G., Cayo A., Cikutović-Molina R., Arevalo B., González W., Catalán M.A., Zúñiga L. (2020). Withaferin A suppresses breast cancer cell proliferation by inhibition of the two-pore domain potassium (K2P9) channel TASK-3. Biomed. Pharmacother..

[294] Bachmann M., Rossa A., Antoniazzi G., Biasutto L., Carrer A., Campagnaro M., Leanza L., Gonczi M., Csernoch L., Paradisi C. (2021). Synthesis and cellular effects of a mitochondria-targeted inhibitor of the two-pore potassium channel TASK-3. Pharmacol. Res..

[295] Czirjak G., Enyedi P. (2003). Ruthenium red inhibits TASK-3 potassium channel by interconnecting glutamate 70 of the two subunits. Mol. Pharmacol..

[296] Antony M.L., Lee J., Hahm E.R., Kim S.H., Marcus A.I., Kumari V., Ji X., Yang Z., Vowell C.L., Wipf P. (2014). Growth arrest by the antitumor steroidal lactone withaferin A in human breast cancer cells is associated with down-regulation and covalent binding at cysteine 303 of beta-tubulin. J. Biol. Chem..

[297] Okamoto S., Tsujioka T., Suemori S.-i., Kida J.-i., Kondo T., Tohyama Y., Tohyama K. (2016). Withaferin A suppresses the growth of myelodysplasia and leukemia cell lines by inhibiting cell cycle progression. Cancer Sci..

[298] Yan Z., Guo R., Gan L., Lau W.B., Cao X., Zhao J., Ma X., Christopher T.A., Lopez B.L., Wang Y. (2018). Withaferin A inhibits apoptosis via activated Akt-mediated inhibition of oxidative stress. Life Sci..

[299] Xia S., Miao Y., Liu S. (2018). Withaferin A induces apoptosis by ROS-dependent mitochondrial dysfunction in human colorectal cancer cells. Biochem. Biophys. Res. Commun..

[300] Chen C.-M., Chung Y.-P., Liu C.-H., Huang K.-T., Guan S.-S., Chiang C.-K., Wu C.-T., Liu S.-H. (2020). Withaferin A protects against endoplasmic reticulum stress-associated apoptosis, inflammation, and fibrosis in the kidney of a mouse model of unilateral ureteral obstruction. Phytomedicine.

[301] Guo R., Gan L., Lau W.B., Yan Z., Xie D., Gao E., Christopher T.A., Lopez B.L., Ma X., Wang Y. (2019). Withaferin A Prevents Myocardial Ischemia/Reperfusion Injury by Upregulating AMP-Activated Protein Kinase-Dependent B-Cell Lymphoma2 Signaling. Circ. J..

[302] Kargacin G.J., Ali Z., Kargacin M.E. (1998). Ruthenium red reduces the Ca^2+^ sensitivity of Ca^2+^ uptake into cardiac sarcoplasmic reticulum. Pflugers Arch..

[303] Xu L., Tripathy A., Pasek D.A., Meissner G. (1999). Ruthenium red modifies the cardiac and skeletal muscle Ca^2+^ release channels (ryanodine receptors) by multiple mechanisms. J. Biol. Chem..

[304] Pope L., Lolicato M., Minor D.L. (2020). Polynuclear Ruthenium Amines Inhibit K(2P) Channels via a "Finger in the Dam" Mechanism. Cell. Chem. Biol..

[305] Kwong J.Q. (2017). The mitochondrial calcium uniporter in the heart: Energetics and beyond. J. Physiol..

[306] Dessi F., Ben-Ari Y., Charriaut-Marlangue C. (1995). Ruthenium red protects against glutamate-induced neuronal death in cerebellar culture. Neurosci. Lett..

[307] Groskreutz J.L., Bronk S.F., Gores G.J. (1992). Ruthenium red delays the onset of cell death during oxidative stress of rat hepatocytes. Gastroenterology.

[308] Zazueta C., Sosa-Torres M.E., Correa F., Garza-Ortiz A. (1999). Inhibitory properties of ruthenium amine complexes on mitochondrial calcium uptake. J. Bioenergy Biomembr..

[309] Zhang L., Hu R., Cheng Y., Wu X., Xi S., Sun Y., Jiang H. (2017). Lidocaine inhibits the proliferation of lung cancer by regulating the expression of GOLT1A. Cell Prolif..

[310] Chang Y.C., Liu C.L., Liu T.P., Yang P.S., Chen M.J., Cheng S.P. (2017). Effect of Perioperative Intravenous Lidocaine Infusion on Acute and Chronic Pain after Breast Surgery: A Meta-Analysis of Randomized Controlled Trials. Pain Pract..

[311] Biel M., Schneider A., Wahl C. (2002). Cardiac HCN channels: Structure, function, and modulation. Trends Cardiovasc. Med..

[312] Santoro B., Tibbs G.R. (1999). The HCN gene family: Molecular basis of the hyperpolarization-activated pacemaker channels. Ann. N. Y. Acad. Sci..

[313] Sartiani L., Mannaioni G., Masi A., Romanelli M.N., Cerbai E. (2017). The Hyperpolarization-Activated Cyclic Nucleotide-Gated Channels: From Biophysics to Pharmacology of a Unique Family of Ion Channels. Pharmacol. Rev..

[314] Zobeiri M., Chaudhary R., Datunashvili M., Heuermann R.J., Luttjohann A., Narayanan V., Balfanz S., Meuth P., Chetkovich D.M., Pape H.C. (2018). Modulation of thalamocortical oscillations by TRIP8b, an auxiliary subunit for HCN channels. Brain Struct. Funct..

[315] Calejo A.I., Reverendo M., Silva V.S., Pereira P.M., Santos M.A., Zorec R., Goncalves P.P. (2014). Differences in the expression pattern of HCN isoforms among mammalian tissues: Sources and implications. Mol. Biol. Rep..

[316] Herrmann S., Layh B., Ludwig A. (2011). Novel insights into the distribution of cardiac HCN channels: An expression study in the mouse heart. J. Mol. Cell. Cardiol..

[317] Lopez-Gonzalez Z., Ayala-Aguilera C., Martinez-Morales F., Galicia-Cruz O., Salvador-Hernandez C., Pedraza-Chaverri J., Medeiros M., Hernandez A.M., Escobar L.I. (2016). Immunolocalization of hyperpolarization-activated cationic HCN1 and HCN3 channels in the rat nephron: Regulation of HCN3 by potassium diets. Histochem. Cell. Biol..

[318] Herrington J., Zhou Y.P., Bugianesi R.M., Dulski P.M., Feng Y., Warren V.A., Smith M.M., Kohler M.G., Garsky V.M., Sanchez M. (2006). Blockers of the delayed-rectifier potassium current in pancreatic beta-cells enhance glucose-dependent insulin secretion. Diabetes.

[319] Xue L., Li Y., Han X., Yao L., Yuan J., Qin W., Liu F., Wang H. (2012). Investigation of hyperpolarization-activated cyclic nucleotide-gated channels in interstitial cells of Cajal of human bladder. Urology.

[320] Luque M., Schrott-Fischer A., Dudas J., Pechriggl E., Brenner E., Rask-Andersen H., Liu W., Glueckert R. (2021). HCN channels in the mammalian cochlea: Expression pattern, subcellular location, and age-dependent changes. J. Neurosci. Res..

[321] León-Aparicio D., Salvador C., Aparicio-Trejo O.E., Briones-Herrera A., Pedraza-Chaverri J., Vaca L., Sampieri A., Padilla-Flores T., López-González Z., León-Contreras J.C. (2019). Novel Potassium Channels in Kidney Mitochondria: The Hyperpolarization-Activated and Cyclic Nucleotide-Gated HCN Channels. Int. J. Mol. Sci..

[322] Gross C., Saponaro A., Santoro B., Moroni A., Thiel G., Hamacher K. (2018). Mechanical transduction of cytoplasmic-to-transmembrane-domain movements in a hyperpolarization-activated cyclic nucleotide-gated cation channel. J. Biol. Chem..

[323] Porro A., Saponaro A., Gasparri F., Bauer D., Gross C., Pisoni M., Abbandonato G., Hamacher K., Santoro B., Thiel G. (2019). The HCN domain couples voltage gating and cAMP response in hyperpolarization-activated cyclic nucleotide-gated channels. eLife.

[324] Fürst O., D’Avanzo N. (2015). Isoform dependent regulation of human HCN channels by cholesterol. Sci. Rep..

[325] Riegelhaupt P.M., Tibbs G.R., Goldstein P.A. (2018). HCN and K2P Channels in Anesthetic Mechanisms Research. Methods Enzymol..

[326] Rivolta I., Binda A., Masi A., DiFrancesco J.C. (2020). Cardiac and neuronal HCN channelopathies. Pflügers Arch.-Eur. J. Physiol..

[327] Santoro B., Shah M.M. (2020). Hyperpolarization-Activated Cyclic Nucleotide-Gated Channels as Drug Targets for Neurological Disorders. Annu. Rev. Pharmacol. Toxicol..

[328] Felix R., Sandoval A., Sanchez D., Gomora J.C., De la Vega-Beltran J.L., Trevino C.L., Darszon A. (2003). ZD7288 inhibits low-threshold Ca^2+^ channel activity and regulates sperm function. Biochem. Biophys. Res. Commun..

[329] Wu X., Liao L., Liu X., Luo F., Yang T., Li C. (2012). Is ZD7288 a selective blocker of hyperpolarization-activated cyclic nucleotide-gated channel currents?. Channnels.

[330] Chevaleyre V., Castillo P.E. (2002). Assessing the role of Ih channels in synaptic transmission and mossy fiber LTP. Proc. Natl. Acad. Sci. USA.

[331] Kleinbongard P., Gedik N., Witting P., Freedman B., Klöcker N., Heusch G. (2015). Pleiotropic, heart rate-independent cardioprotection by ivabradine. Br. J. Pharmacol..

[332] Postea O., Biel M. (2011). Exploring HCN channels as novel drug targets. Nat. Rev. Drug Discov..

[333] Goto M., Miyahara I., Hirotsu K., Conway M., Yennawar N., Islam M.M., Hutson S.M. (2005). Structural determinants for branched-chain aminotransferase isozyme-specific inhibition by the anticonvulsant drug gabapentin. J. Biol. Chem..

[334] Pielen A., Kirsch M., Hofmann H.D., Feuerstein T.J., Lagreze W.A. (2004). Retinal ganglion cell survival is enhanced by gabapentin-lactam in vitro: Evidence for involvement of mitochondrial K_ATP_ channels. Graefes Arch. Clin. Exp. Ophthalmol..

[335] Hainsworth A.H., McNaughton N.C., Pereverzev A., Schneider T., Randall A.D. (2003). Actions of sipatrigine, 202W92 and lamotrigine on R-type and T-type Ca^2+^ channel currents. Eur. J. Pharmacol..

[336] Kranzler H.R., Feinn R., Morris P., Hartwell E.E. (2019). A meta-analysis of the efficacy of gabapentin for treating alcohol use disorder. Addiction.

